# Recent and projected changes in climate patterns in the Middle East and North Africa (MENA) region

**DOI:** 10.1038/s41598-024-60976-w

**Published:** 2024-05-04

**Authors:** Diana Francis, Ricardo Fonseca

**Affiliations:** Environmental and Geophysical Sciences (ENGEOS) Lab, Earth Sciences Department, Khalifa University of Science and Technology, P. O. Box 127788, Abu Dhabi, United Arab Emirates

**Keywords:** Heat low, Tropics expansion, Global warming, Trends, CMIP6, MENA region, Climate sciences, Atmospheric science, Climate change

## Abstract

Observational and reanalysis datasets reveal a northward shift of the convective regions over northern Africa in summer and an eastward shift in winter in the last four decades, with the changes in the location and intensity of the thermal lows and subtropical highs also modulating the dust loading and cloud cover over the Middle East and North Africa region. A multi-model ensemble from ten models of the Coupled Model Intercomparison Project—sixth phase gives skillful simulations when compared to in-situ measurements and generally captures the trends in the ERA-5 data over the historical period. For the most extreme climate change scenario and towards the end of the twenty-first century, the subtropical highs are projected to migrate poleward by 1.5°, consistent with the projected expansion of the Hadley Cells, with a weakening of the tropical easterly jet in the summer by up to a third and a strengthening of the subtropical jet in winter typically by 10% except over the eastern Mediterranean where the storm track is projected to shift polewards. The length of the seasons is projected to remain about the same, suggesting the warming is likely to be felt uniformly throughout the year.

## Introduction

The Earth’s climate has changed considerably over time^1,2^, and more recently since the Industrial Revolution in the late eighteenth century due to increased anthropogenic emissions^3,4^. Such changes have been felt particularly strongly in the arid and semi-arid regions of the Middle East and Northern Africa (MENA^5–8^), and are likely to continue in a warming climate^9^, with the rapid population growth further increasing the societal impacts of higher anthropogenic emissions^10^. In-situ ground-based observations^11–13^ and those collected by remote sensing assets^14,15^, confirm these changes.

The MENA region is one of the most water scarce regions in the world, being particularly vulnerable to climate change^10,16^. The spatially-averaged annual air temperature here has increased at a rate of 0.36 °C/decade during 1981–2020, with an even more marked increase of 0.45 °C/decade in the summer^17^. Temperature extremes have also become more frequent^18,19^: e.g. the July 2023 heatwave brought temperatures of up 51 °C in Algeria, 49 °C in Tunisia and 46 °C in Jordan, leading to widespread power outages and forest fires^20^. Model projections for the “business-as-usual” climate change scenario indicate that half of the population in the MENA region (roughly 600 million people) could be exposed to recurring super- and ultra-extreme heatwaves, which will feature air temperatures up to 56 °C and higher lasting for several weeks at a time, in the second half of this century^21^. Even though the aridity in the MENA region has significantly increased in recent decades^8^, extreme rainfall events may be more impactful in a warming world^22,23^. In Jeddah, on the western coast of Saudi Arabia, extreme precipitation events are becoming less frequent but more intense, with a shift towards a larger effect of extratropical forcing over tropical forcing in recent decades^24^. In September 2023, and owing to the high sea surface temperatures (SSTs) and advection of cooler air at upper-levels in the atmosphere, storm Daniel developed in the Mediterranean Sea, wreaking havoc in particular in Greece and Libya where it took the lives of more than 5000 people^25^. As the sea surface warms, the increased evaporation and moisture content of the atmosphere will promote more extreme precipitation events^26^. Higher elevation regions may experience an even more marked increase in rainfall extremes at a rate of about 15% per degree Celsius of warming, roughly doubled that expected from the increase in water vapour as given by the Clausius-Clapeyron relationship, driven by a shift in the snow-rain partitioning^27^.

Even though the MENA region is particularly sensitive to a changing climate, only a limited number of studies have been published on the recent meteorological changes here, and have yielded mixed results, failing to provide a clear picture of the evolution in the last few decades. For example, there is little agreement in terms of the trend in the 10 m wind speed between the different reanalyses over North Africa and the Arabian Peninsula, while over the Sahel, India and Pakistan they generally exhibit a downward trend in winter for 1980–2015^28^. Reanalysis and observational datasets exhibit a warming and drying tendency over the Middle East and the Mediterranean, albeit with different magnitudes^29^. Zhou et al. ^30^ investigated trends in dust loading from the Modern-Era Retrospective analysis for Research and Applications version 2 (MERRA-2^31^) reanalysis dataset from 1980 to 2020. They found an increase in dust in arid/semi-arid regions of mid-/high-latitudes outside dust source regions driven by higher temperatures, which decrease the atmospheric stability and promote dust lifting. The integrated water vapour amounts have generally been decreasing over northeastern Africa and the Arabian Peninsula in winter but increasing in the summer, owing to higher evaporation levels from the adjacent warmer water bodies^32^. On top of the background trend there is considerable variability on inter-annual and decadal time-scales, seen in fields such as temperature^33,34^, precipitation^35,36^, dust loading^37,38^ and cloud cover^39,40^.

As moisture, clouds and dust greatly impact the radiation fields, a proper understanding of the long-term trends in observational and reanalysis data requires an investigation of the circulation and the atmospheric patterns, which modulate those fields. Besides solar energy applications^41^, a better evaluation of the different components of the surface energy budget is a necessary step to improve our current understanding of the effects of aerosols and clouds on the atmosphere and ocean^42,43^, as well as the complex soil-atmosphere interactions in particular in arid and semi-arid regions where the water table can be quite shallow^44,45^. This has not been accomplished so far in the MENA region. As the spatial scales of the major atmospheric drivers exceed those of the MENA region, an in order to have a better understanding of the past and projected changes in the region, in this study the analysis will be conducted over the extended MENA region, which comprises northern and equatorial Africa, the Middle East, southwest Asia and southern Europe. This is crucial given the high sensitivity of the region to climate change^8^ and that, in some parts, the combination of heat and humidity has already crossed a threshold for human habitability, a tendency that will likely be exacerbated in a warming climate^46^.

Climate change projections for the MENA region generally point towards a warmer and drier climate with increased extreme events by the end of the twenty-first century^47,48^, although by varying magnitudes^49^. reported that, according to the Coupled Model Intercomparison Project—sixth phase (CMIP6^50^) model projections, by the end of the twenty-first century there will be widespread warming over Africa more pronounced over the Sahara, with reduced precipitation amounts over northern and southern parts of the continent and increased in central parts, suggesting a broadening of the Hadley Cells. The median warming in the CMIP6 Multi-Model Ensemble Mean (MMEM) is found to be higher than that in the Coupled Model Intercomparison Project—fifth phase (CMIP5^51^) MMEM over most of Africa, reaching up to 2.5 K over some regions, with a mixed pattern in terms of precipitation. Chen et al.^52^ showed the extremes in CMIP6 are generally an improvement with respect to those given by the models that integrated CMIP5, in particular for the annual maximum of the daily maximum and annual minimum of the daily minimum air temperatures.

Coupled with increased sea surface temperatures and surface evaporation leading to higher atmospheric moisture levels, future wet-bulb temperatures over Southwest Asia are projected to exceed a threshold for human habitability^53^. To date, most of the work conducted on the analysis of CMIP6 model data has focused on selected variables, in particular mean and extreme temperature and precipitation, with less attention paid to circulation patterns and seasonal changes, which impact regional and local conditions. For example, a change in the position and/or strength of the thermal (heat) lows and subtropical highs over the Middle East and North Africa will impact the atmospheric circulation and wind patterns locally^54,55^. This can in turn impact the transport of moisture^56^ and the dust/aerosol emissions and transport^57,58^ and hence the atmospheric radiation fields^59^. Such circulation changes in a warming climate have not been comprehensively discussed, and will be explored here for a CMIP6 MME for the extended MENA region.

This study has three main objectives: (1) Identify the trends in regional climate patterns over Northern Africa, the Middle East, southern Europe and southwest Asia based on observational and reanalysis products over the last four decades, linking them to changes in different parameters such as temperature, clouds, moisture and radiation; (2) Assess how well these trends are represented in a CMIP6 MME accounting for the inter-model spread; (3) Highlight the main projected changes in the atmospheric circulation and in the length of the seasons as given by the MME for the most extreme climate change scenario, the Shared Socioeconomic Pathway SSP5-8.5 scenario. SSP5-8.5 corresponds to a case in which mitigation challenges dominate over adaptation ones, with an expected radiative forcing of 8.5 W m^−2^ by 2100^60,61^. The findings of this work will help to further our understanding on how the atmospheric circulation has evolved in the recent past and is projected to change in the future in a region that comprises the world’s largest deserts and where the population continues to grow at a very fast pace^62^. An increased population and higher sensitivity to a changing climate further exacerbates the impacts of climate change on peoples’ lives and stresses the need for an accurate knowledge of how the climate has changed and is projected to continue to do so. This is needed for the development of adaptation and mitigation strategies for the infrastructure^46^, agriculture^63^ and human health^64^. On the other hand, the usage of renewable energy sources in the MENA region has increased exponentially in the last few decades, in particular solar energy^65,66^ a trend expected to further accelerate in the future. Hence, it is crucial to gain knowledge on the region’s evolving climate in order to optimize the design and operational strategies for such alternative energy sources^67^.

## Data and methods

### Observational and reanalysis datasets

Three satellite-derived and two reanalysis gridded products are used in this study, in addition to weather station (in situ) observations in the target domain (30° W–80° E, 15° S–45° N), an extended MENA region that covers northern and equatorial Africa, the Middle East, southern Europe and southwest Asia.

The amount of water vapour in the atmospheric column, also known as precipitable water, is estimated from the Morphed Integrated Microwave Imagery at the Cooperative Institute for Meteorological Satellite Studies—Total Precipitable Water version 2 (MIMIC-TPW2) dataset^68^. This product, which makes use of measurements from several operational microwave-frequency sensors such as the Special Sensor Microwave Imager Sounder (SSMI/S) and the Advanced Microwave Scanning Radiometer-Earth Observing System (AMSR-E), provides hourly global precipitable water maps from October 2016 to present on a 0.25° × 0.25° (~ 27 km spatial resolution) grid. The mean average error of this dataset is estimated to be of about 0.5–2 mm^68^ and hence this product is suitable for the trend analysis conducted here.

Cloud cover and cloud properties are given by the European Organisation for the Exploitation of Meteorological Satellites (EUMETSAT) Climate Monitoring Satellite Application Facility (CM SAF) Cloud, Albedo and Surface Radiation dataset produced from the measurements collected by the Advanced Very High Resolution Radiometer (AVHRR) data—second edition (CLARA-A2^69^). Fields available on a 0.25° × 0.25° (~ 27 km) grid from 1982 to present include the daily-mean total cloud cover; cloud fraction associated with low (clouds below 680 hPa), middle (680 hPa and 440 hPa) and high-level (above 440 hPa) clouds; daytime (solar zenith angle less than 75°) and nighttime (solar zenith angle higher than 95°) cloud fraction; cloud top temperature, pressure and height. The bias in the cloud fraction products in the MENA region is mostly in the range ± 5%^70^ which is a good skill given the large temporal and spatial variability of cloud cover in this region. In fact^39^, assessed the performance of different cloud products over the Arabian Peninsula between 1984 and 2009 and found that CLARA has the lowest bias with respect to ground observations, even though it tends to report false clouds in arid and semi-arid regions likely due to the heterogeneous land surface emissivity.

Surface radiation fluxes are given by the Clouds and the Earth’s Radiant Energy System (CERES^71^) dataset. Upward and downward shortwave and longwave fluxes are available hourly on a 1° × 1° (~ 111 km) resolution from the SYN1deg—Level 3 product from March 2000 to present. The biases in the CERES fluxes in the MENA region are in the range ± 5%, with the timing of the daily maximum in the shortwave flux and the daily maximum and minimum in the longwave flux well captured^72,73.^

Precipitation estimates are extracted from the measurements collected by the Global Precipitation Measurement (GPM) satellite, a joint mission between the United States’ National Aeronautic and Space Administration (NASA) and the Japanese Aerospace and Exploration Agency (JAXA), available on a 0.1° × 0.1° grid and on a temporal resolution ranging from 30 min to monthly (the daily data is used). Version 7, the latest version, is available from June 2000 to present. The Integrated Multi-Satellite Retrievals for GPM (IMERG) product has a good performance in the MENA region, with a probability of detection generally above 0.6 and a false alarm rate below 0.4^74^. A comparison with rain gauge measurements in the UAE, a country that is representative of the hyper-arid conditions in the MENA region, revealed root mean squared errors generally within 20 mm and correlations in excess of 0.5 over the vast majority of the country^75^.

The two reanalysis datasets considered are MERRA-2^31^ and ERA-5^76^. In particular, ERA-5 is the main reanalysis product considered, with MERRA-2 used solely for the dust optical depth. The MERRA-2 reanalysis, available hourly on a 0.5° × 0.625° grid from 1980 to present, explicitly represents aerosols and their interactions with the climate system. It assimilates the aerosol optical depth (AOD) from the AVHRR and the Moderate Resolution Imaging Spectroradiometer (MODIS) instruments, the Multiangle Imaging Spectroradiometer (MISR) over bright surfaces, and the Aerosol Robotic Network (AERONET) sunphotometer stations^77^. MERRA-2 is found to perform well when compared to in situ measurements over the Middle East^78^, northern Africa and Southern Europe^79,80^. In particular, the total AOD in this product is mostly within 0.1 of that observed from ground-based observations in the MENA region^81^. Hourly dust AOD is downloaded for the period 1980–2022 and subsequently used for the trend analysis. ERA-5 is the latest reanalysis dataset produced by the ECMWF, and is available on an hourly basis from 1940 to present and on a 0.25° × 0.25° grid. It is regarded as one of the best performing reanalysis datasets globally, including over the target domain, in terms of surface fluxes^82,83^, precipitation, including trends^84,85^, and temperature^86^. Hourly air and dewpoint temperature, surface pressure, sea-level pressure, horizontal wind components and precipitation are extracted from January 1980 to December 2022. In order to compare with the CMIP6 daily maximum and minimum temperatures, the ERA-5 daily maximum and minimum temperatures are estimated as the highest and lowest temperatures in a given day from 00 to 23 UTC.

Weather station data is available from the National Centers for Environmental Information Global Surface Summary of the Day (GSOD^87^) dataset. It contains daily statistics from more than 9000 weather stations worldwide from 1929 to present, including daily-mean air temperature, dewpoint temperature, sea-level pressure, visibility, wind speed, daily precipitation amount and daily minimum and maximum temperature. A total of 574 weather stations in the extended MENA region that have continuous coverage in the 35-year period 1980–2014 are considered in this work. A strict and extensive quality control procedure is applied to the data, yielding a very small uncertainty^88^.

### CMIP6 models

Besides the observational and reanalysis datasets, daily and monthly data of ten models that are part of CMIP6 for the historical period (1980–2014) and the SSP5-8.5 climate change scenario (2015–2100) are downloaded from the World Climate Research Programme website^89^. Two criteria are considered when selecting the models: (1) the nominal resolution is no lower than 100 km, as a higher spatial resolution is needed to properly represent the complex land-sea mask and topography in the target region; (2) data is available for both the historical and climate change periods for all selected variables. For daily fields, the latter include the daily-mean air temperature, wind speed, sea-level pressure, specific humidity, daily precipitation and daily minimum and maximum air temperatures. For the monthly fields, the variables considered are the zonal and meridional wind components and geopotential height on 19 pressure levels. The characteristics of the models selected are given in Tables 1, 2. HadGEM3-GC31-MM data is not used for daily data as it does not follow the Gregorian calendar (all months have 30 days).

In order to construct the multi-model ensemble (MME), the daily and monthly data from all 10 models and realizations (i.e. members) are bi-linearly interpolated to the grid of the coarsest of all, which is 2° × 1.5°, before doing the averaging and extracting the standard deviation regarded as a measure of the uncertainty. The model simulations are first assessed against the NOAA GSOD measurements and the trends compared to those given by ERA-5 before being used to explore the climate change projections for the SSP5-8.5 scenario. For analysis, the climate change period of 2066–2100 is selected as it comprises 35 years, the same number of years as the historical period (1980–2014), and is towards the end of the twenty-first century when the changes in atmospheric dynamics and thermodynamics are projected to be more extreme.

As different models have a different number of realizations, traditionally weights are assigned to each, with the most common approaches being one model one vote or applying weights that account for the model performance in comparison with observations. As noted by^90^, the former is a safer and more transparent way to combine models. For models with more than one realization, authors have either assigned a weight of 1/*N* to each realization with *N* being the number of realizations for that particular model^91^, or selected one realization for each model, e.g. the first one^92^, or averaged over all realizations^93^. In some studies, no weights are applied^94^. In this case, each realization of each model is regarded as an ensemble member, carrying the same weight as all others. The latter approach is used in this work, as it is found to give the best agreement with NOAA GSOD station data in the MENA region compared to assigning a 1/N weight to each realization of a given model, or taking the mean or the median of all realizations or selecting the first realization of a given model to ensure a one model one vote approach (not shown). It is also important to note that, as seen in Tables 1, 2, for 60–90% of the models there are only 1–3 realizations, with only 2–3 models having 10–11 realizations, and hence the question of how to handle different realizations of a given model is not as pertinent for the CMIP6 MME constructed in this work as in other studies where a much higher number of ensemble members is considered.

**Table 1:** CMIP6 models for which daily data is considered in this study and their characteristics.

CMIP6 model	Resolution	Ensemble members		References
		Historical	SSP5-8.5	
BCC-CSM2-MR (Beijing Climate Center—Climate System Model version 2—Medium Resolution)	1.125° × 1.125° × 46L	2	1	^95^
CMCC-ESM2 (Centro Euro-Mediterraneo per I Cambiamenti Climatici—Earth System Model version 2)	1.25° × 0.9° × 30L	1	1	^96^
*GFDL-CM4* (Geophysical Fluid Dynamics Laboratory—Global Climate Model version 4)	1.25° × 1° × 33L	1	1	^97^
GFDL-ESM4 (Geophysical Fluid Dynamics Laboratory—Earth System Model version 4)	1.25° × 1° × 49L	3	1	^98^
INM-CM4-8 (Institute for Numerical Mathematics—Coupled Model version 4.8)	2° × 1.5° × 21L	1	1	^99^
INM-CM5-0 (INM—Coupled Model version 5.0)	2° × 1.5° × 73L	10	1	^100^
MPI-ESM1.2-HR (Max Planck Institute for Meteorology—Earth System Model version 1.2—High Resolution)	0.9° × 0.9° × 95L	10	2	^101^
MRI-ESM2-0 (Meteorological Research Institute—Earth System Model version 2.0)	1.1° × 1.1° × 80L	11	6	^102^
NorESM2-MM (Norwegian Earth System Model version 2.0 with moderate resolution for atmosphere, land and ocean)	1.25° × 0.9° × 32L	3	1	^103^
TaiESM1 (Taiwan Earth System Model version 1)	1.25° × 0.9° × 30L	1	1	^104^

**Table 2:** CMIP6 models for which monthly data is considered in this study and their characteristics.

CMIP6 model	Resolution	Ensemble Members		References
		Historical	SSP5-8.5	
BCC-CSM2-MR (Beijing Climate Center—Climate System Model version 2—Medium Resolution)	1.125° × 1.125° × 46L	3	1	^95^
CMCC-ESM2 (Centro Euro-Mediterraneo per I Cambiamenti Climatici—Earth System Model version 2)	1.25° × 0.9° × 30L	1	1	^96^
GFDL-ESM4 (Geophysical Fluid Dynamics Laboratory—Earth System Model version 4)	1.25° × 1° × 49L	3	1	^98^
HadGEM3-GC31-MM (Hadley Center Global Environmental Model—Global Coupled 3.1 with medium atmosphere and ocean resolution)	0.8° × 0.6° × 85L	4	4	^105^
INM-CM4-8 (Institute for Numerical Mathematics—Coupled Model version 4.8)	2° × 1.5° × 21L	1	1	^99^
INM-CM5-0 (INM—Coupled Model version 5.0)	2° × 1.5° × 73L	10	1	^100^
MPI-ESM1.2-HR (Max Planck Institute for Meteorology—Earth System Model version 1.2—High Resolution)	0.9° × 0.9° × 95L	10	2	^101^
MRI-ESM2-0 (Meteorological Research Institute—Earth System Model version 2.0)	1.1° × 1.1° × 80L	11	5	^102^
NorESM2-MM (Norwegian Earth System Model version 2.0 with moderate resolution for atmosphere, land and ocean)	1.25° × 0.9° × 32L	3	1	^103^
TaiESM1 (Taiwan Earth System Model version 1)	1.25° × 0.9° × 30L	2	1	^104^

### Diagnostics and metrics

The performance of the CMIP6 MME is evaluated against the in-situ measurements collected at the GSOD stations using the verification diagnostics proposed by^106^. In particular, the bias, the correlation $\rho$, the variance similarity η and the normalized error variance α are considered. They are defined in Eqs. (1–4) below, where **F** is the forecast or simulation, **O** is the observations, **D** is the discrepancy between the two, and $<\mathbf{X}>$ and ${}{\varvec{X}}$ are the mean and standard deviation of $\mathbf{X}$, respectively. The bias is given by the mean discrepancy between the model simulations and observations. The correlation $\rho$ is a measure of the phase agreement between the model simulations and observations, while the variance similarity $\eta$ indicates the amplitude agreement between the two. The normalized error variance $\alpha$ is the variance of the error arising from discrepancies between the observed and modeled phase and amplitude, normalized by the combined modeled and observed signal variances. The best performance corresponds to a bias of 0, $\rho$ = $\eta$ = 1 and $\alpha$ = 0. For a random simulation $\alpha$ = 1, and hence for a model simulation to be deemed useful $\alpha$ < 1. The diagnostics $\rho$, $\eta$ and $\alpha$ are non-dimensional, symmetric with respect to simulations and observations, and can be extracted for scalar and vector variables. These diagnostics are computed both over time and space. In the first approach, and at the location of each station and for a given season, the skill scores are extracted using all the days in the historical period (1980–2014). In the second approach, and for a given season and each day, the skill scores are computed using data from all the stations. Box plots are then constructed to highlight the range of values obtained for the stations and time-period considered, respectively, for a given season.

1 $$BIAS = \langle\varvec{D}\rangle = \langle\varvec{F}\rangle - \langle\varvec{O}\rangle$$

2 $$\rho = \frac{1}{{\sigma _{O} \sigma _{F} }}\langle\left( {\varvec{F} - \langle\varvec{F}\rangle} \right) \cdot \left( {\varvec{O} - \langle\varvec{O}\rangle} \right)\rangle,~ - 1 \le \rho \le 1$$

3 $$\eta = \frac{{\sigma_{O} \sigma_{F} }}{{\frac{1}{2}\left( {\sigma_{O}^{2} + \sigma_{F}^{2} } \right)}}, 0 \le \eta \le 1$$

4 $$\alpha = 1 - \rho \eta = \frac{{\sigma_{D}^{2} }}{{\sigma_{O}^{2} + \sigma_{F}^{2} }}, 0 \le \alpha \le 2$$

The trend and intercept of the linear regression are obtained with the Theil-Sen estimator, which is a non-parametric test that is insensitive to outliers and more robust than the ordinary least square regression technique ^107,108^. The statistical significance is assessed with the Mann–Kendall test^109^ considering a confidence level of 95%.

Changes in the length of the seasons are inferred with the percentile metric described in^110^ applied to the daily-mean air temperature. First, the 29 February is excluded in leap years so all years have 365 days, and the number of years selected for the historical (1980–2014) and climate change (2066–2100) periods is also the same, 35-years in each, in order to ensure a fair comparison. For each year, summer is defined as the period when the daily-mean air temperature is above the 75th percentile for that year, and winter when it is below the 25th percentile. Spring and autumn are the transition seasons with the temperature increasing in the former and decreasing in the latter. As in^110^, and in order to smooth the high frequency variability in the daily-mean temperature data and avoid more than two intersections with each temperature threshold, a third-degree polynomial function is fitted to the raw data. This methodology, applied here to each grid-point in the domain (i.e. the temperature thresholds are spatially varying), has the advantage of being insensitive to the background warming, which would have resulted in a shorter winter and longer summer season in a future climate. This is not true for other techniques such as the seasonal shift metric proposed by^111^. Furthermore, the statistical significance of the projected changes in the seasons’ lengths is assessed with the bootstrap technique considering 1000 samples^112^.

An important feature of the large-scale circulation in the target region is the subtropical high, which is typically diagnosed based on the maximum geopotential height at a selected pressure level such as 850 hPa, 500 hPa or 300 hPa^113,114^. Here, the position of the subtropical high over North Africa (0°–35° N; 15° W–30° E) and Arabian Peninsula (5°–35° N; 35°–60° E) is identified based on the maximum of the 500 hPa geopotential height over each area, as this standard pressure level is above the complex topography in both regions. The bootstrapping technique^112^ is used to assess whether the projected changes in the position of each subtropical high from the historical to the climate change period are statistically significant at 95% confidence level considering 1000 samples.

The position and strength of the Saharan Heat Low (SHL^115^) and Arabian Heat Low (AHL^116^) are characterized using the low-level atmospheric thickness (LLAT) metric, given by the difference between the 700 hPa and 925 hPa geopotential height values. In particular, the SHL corresponds to the area with the 10% highest LLAT in the region 20° W–30 °E and 0°–40° N at 06 UTC, just before local sunrise, as during daytime this field is impacted by the complex surface albedo pattern^115^. For the AHL, the LLAT is taken at 03 UTC and in the region 40°–60° E and 10°–35° N, excluding both water bodies and areas where the 925 hPa pressure surface is below topography^116^. For each heat low, the intensity is given by the average LLAT value over the defined heat low regions, while the latitude/longitude is defined as the LLAT-weighted latitude/longitude. Following^116^, the Intertropical Discontinuity (ITD), which is the boundary between the hotter and drier desert air and the cooler and more moist air over the Gulf of Guinea/Arabian Sea, is diagnosed using the 15 °C isoline of dewpoint temperature over West Africa (10°W–20° E) and the Arabian Peninsula (43°–60° E).

## Results and discussion

### Trends in observational and reanalysis data

**Figure 1:**
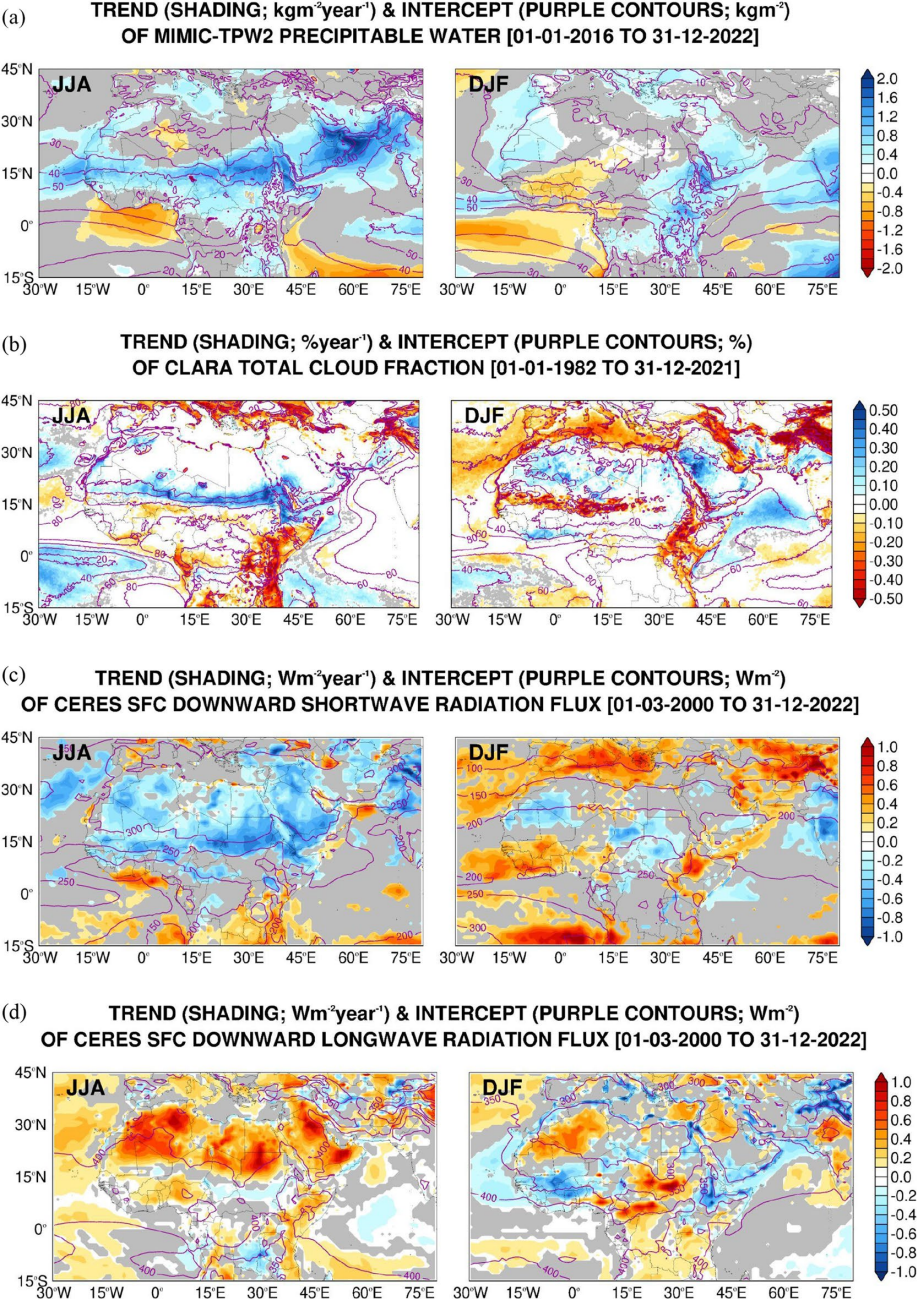
Linear regression of observational datasets: (**a**) Intercept (solid purple contours; kg m^−2^) and trend (shading; kg m^−2^ year^−1^) of daily-mean precipitable water as given by the MIMIC-TPW2 dataset from 01 October 2016 to 31 December 2022 for the boreal summer (JJA) and winter (DJF) seasons. Regions where the trend is not statistically significant at 95% confidence level are shaded in grey. The intercept and slope are obtained with the Theil-Sen estimator while the Mann–Kendall test is used for statistical significance. (**b**–**d**) are as (**a**) but for the daily-mean cloud cover from CLARA (units of % and % year^−1^, respectively) from 01 January 1982 to 31 December 2021, and the daily-mean surface downward short-wave and long-wave radiation flux (units of W m^−2^ and W m^−2^ year^−1^) from CERES for 01 March 2000 to 31 December 2022, respectively.

**Figure 2:**
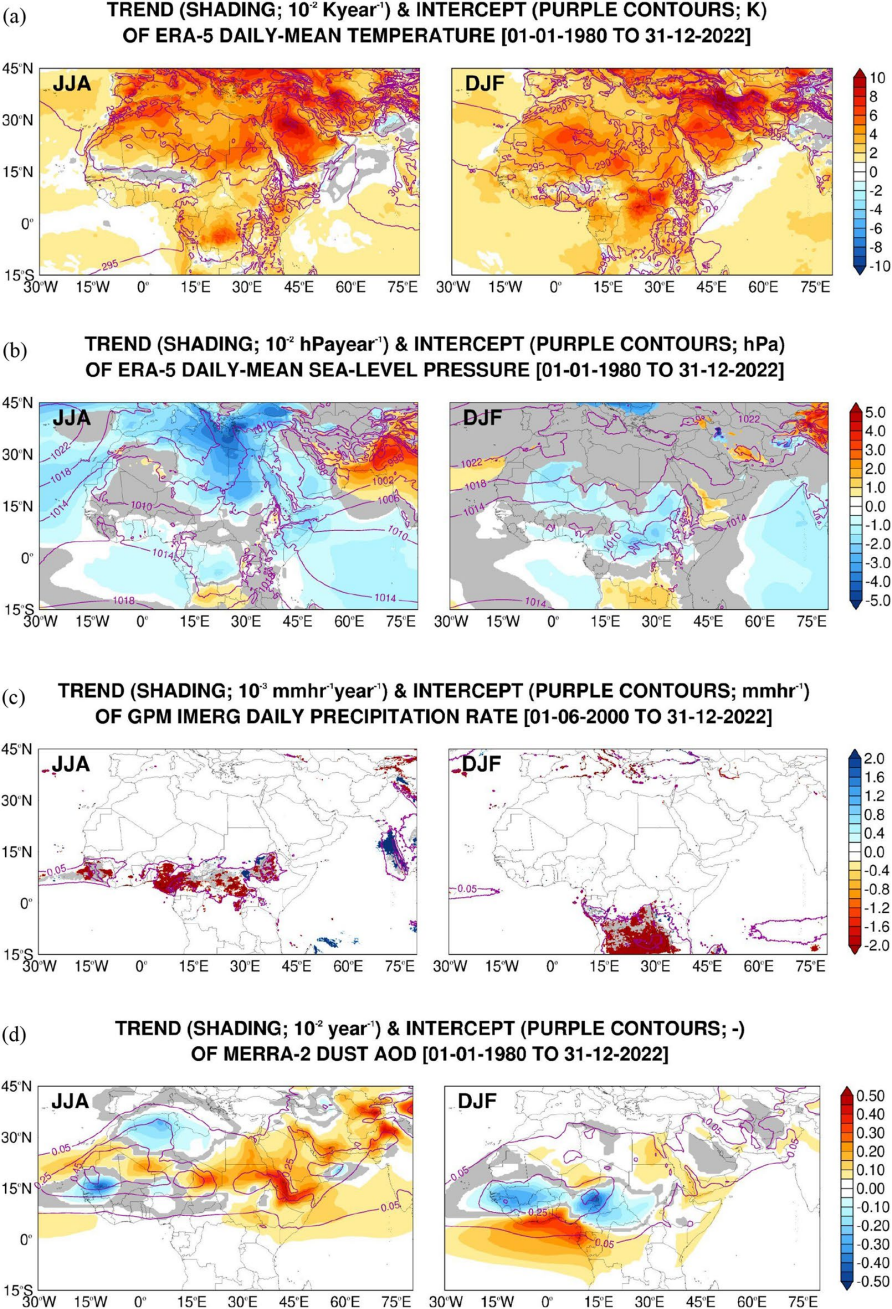
Linear regression of reanalysis datasets: As Fig. 1 but for the daily-mean ERA-5 (**a**) air temperature (units of K and 10^−2^ K year^−1^ for the intercept and trend, respectively) and (**b**) sea-level pressure (shading; hPa and 10^−2^ hPa year^−1^) for the period 01 January 1980 to 31 December 2022; (**c**) GPM IMERG daily precipitation rate (mm h^−1^ and 10^−3^ mm h^−1^ year^−1^) for the period 01 June 2000 to 31 December 2022; and (**d**) MERRA-2 dust aerosol optical depth (AOD; non-dimensional and 10^−2^ year^−1^ for the period 01 January 1980 to 31 December 2022.

Linear trends in selected observational (Fig. 1) and reanalysis (Fig. 2) fields for the summer (June to August, JJA) and winter (December to February, DJF) seasons and for the years when data is available are investigated. The values of the intercept and slope for the MENA region (13°W-60°E, 15°-40°N) are given in Table 3.

**Table 3:** Mean and standard deviation of the intercept and slope for summer (top) and winter (bottom) seasons of several fields for the MENA region (13° W–60° E, 15°–40° N).

Variable	Seasons	Intercept	Trend
Precipitable water	Summer	23.437 ± 7.487 kg m^−2^	0.633 ± 0.484 kg m^−2^ year^−1^
	Winter	11.461 ± 4.272 kg m^−2^	0.248 ± 0.272 kg m^−2^ year^−1^
Total cloud fraction	Summer	14.853 ± 16.313%	0.037 ± 0.119% year^−1^
	Winter	37.559 ± 22.726%	− 0.028 ± 0.171% year^−1^
Low cloud fraction	Summer	2.805 ± 6.198%	0.00005 ± 0.034% year^−1^
	Winter	7.875 ± 6.277%	0.066 ± 0.096% year^−1^
Middle cloud fraction	Summer	1.174 ± 2.505%	0.002 ± 0.020% year^−1^
	Winter	5.809 ± 7.958%	− 0.010 ± 0.050% year^−1^
High cloud fraction	Summer	2.730 ± 7.056%	0.017 ± 0.060% year^−1^
	Winter	1.527 ± 3.366%	0.000 ± 0.000% year^−1^
Daytime cloud fraction	Summer	10.753 ± 18.259%	0.008 ± 0.087% year^−1^
	Winter	30.524 ± 27.584%	0.001 ± 0.092% year^−1^
Nighttime cloud fraction	Summer	14.170 ± 20.073%	0.014 ± 0.126% year^−1^
	Winter	37.594 ± 26.991%	− 0.073 ± 0.182% year^−1^
Cloud top temperature	Summer	277.304 ± 10.882 K	0.007 ± 0.225 K year^−1^
	Winter	268.803 ± 8.705 K	0.154 ± 0.123 K year^−1^
Downward SW radiation flux	Summer	314.676 ± 20.204 W m^−2^	− 0.326 ± 0.252 W m^−2^ year^−1^
	Winter	166.996 ± 53.016 W m^−2^	0.169 ± 0.367 W m^−2^ year^−1^
Downward LW radiation flux	Summer	391.378 ± 23.860 W m^−2^	0.337 ± 0.287 W m^−2^ year^−1^
	Winter	307.890 ± 29.411 W m^−2^	− 0.013 ± 0.393 W m^−2^ year^−1^
Air temperature	Summer	302.401 ± 4.748 K	0.044 ± 0.018 K year^−1^
	Winter	286.304 ± 5.938 K	0.042 ± 0.020 K year^−1^
Sea-level pressure	Summer	1009.23 ± 4.890 hPa	− 0.016 ± 0.011 hPa year^−1^
	Winter	1016.79 ± 2.349 hPa	− 0.008 ± 0.011 hPa year^−1^
Precipitation rate	Summer	7.7 × 10^−5^ ± 0.001 mm h^−1^	4.4 × 10^−8^ ± 7.1 × 10^−6^ mm h^−1^ year^−1^
	Winter	0.0014 ± 0.005 mm h^−1^	− 2.1 × 10^−6^ ± 4.3 × 10^−5^ mm h^−1^ year^−1^
Dust optical depth	Summer	0.232 ± 0.160	0.0006 ± 0.0012 year^−1^
	Winter	0.072 ± 0.068	0.0003 ± 0.0004 year^−1^

Only grid-points for which the trends are statistically significant at the 95% confidence interval within the MENA domain are considered to generate the statistics.

The intercepts show the background atmospheric conditions in the region. Of note is the Intertropical Convergence Zone (ITCZ), associated with higher levels of moisture (Fig. 1a; precipitable water values in excess of 50 kg m^−2^), cloud cover (Fig. 1b; above 80%) and precipitation (Fig. 2c; in excess of 0.1 mm h^−1^) and cooler air temperatures (Fig. 2a; 295–305 K) compared to the neighboring regions, migrating northwards in JJA and southwards in DJF. Its signature is also present in the surface radiation fields (Fig. 1c,d), as evidenced by the reduced surface downward shortwave and increased downward longwave radiation fluxes, typically by 50–100 W m^−2^. The SHL and the AHL are the main low-level circulation features in the summer (Fig. 2b), which develop in response to the strong surface heating of the correspondent land areas (Fig. 2a). The meridional pressure gradient over northern Africa between the thermal low to the south and the subtropical high to the north gives rise to easterly to northeasterly winds that trigger dust emission ^54,57^which is subsequently transported downwind into the Atlantic Ocean (Fig. 2d). Cut-off lows and dry cyclones also lead to dust lofting^117–120^.

The presence of dust also impacts the surface radiation fields (Fig. 1c–e,d), in particular the longwave radiation flux. In the Arabian Peninsula, dust emission is mostly associated with the Shamal winds, which arise from the pressure gradient between the subtropical high over northern Saudi Arabia and the eastern Mediterranean and the monsoon trough^55,121–123^. Dry cyclones^43,124^ and cut-off lows^58^ are also important dust lifting mechanisms here. In winter, the Siberian anticyclone over Asia, and the subtropical high over the northeastern Atlantic extending into northern Africa, with central pressures in excess of 1020 hPa, exert the largest control, with the African thermal low shifting equatorwards^115^ (Fig. 2b). In both North Africa and the Arabian Peninsula, the dust emission is reduced in the colder months (Fig. 2d), but it does occur, mostly in association with mid-latitude weather systems^122,125,126^.

Having described the background state of the different variables and highlighted the major dynamic and thermodynamic features in the MENA region, the trends are now discussed. The trend of precipitable water (Fig. 1a) indicates an increased moistening of the northern Arabian Sea and southeastern parts of the Arabian Peninsula and a poleward shift of the ITCZ over northern Africa in the summer, with a mean value of 0.6 kg m^−2^ year^−1^ in the MENA region (Table 3). These tendencies are also seen in the precipitation (Fig. 2c), cloud cover (Fig. 1b) and surface radiation fields (Fig. 1c–d). The northward migration of the ITCZ is thought to be at least partially driven by the recent warming in the Mediterranean and Saharan regions^127^. This is seen in the air temperature trend (Fig. 2a), which features widespread positive values except mainly in regions of increased rainfall such as along the ITCZ and over northwestern India and Pakistan.

A northward shift of the Somali Jet in response to the enhanced meridional temperature gradient between the Middle East and the southern Arabian Sea owing to a stronger surface heating (and resulting lower surface pressures) over the Middle East is also seen in spring, as highlighted in^128^. In the MENA region in particular, the air temperature has been increasing at a rate of about 0.04 K year^−1^ and the sea-level pressure has been dropping at about 0.016 hPa year^−1^ (Table 3), trends that are steeper than those seen elsewhere (Fig. 2a,b). The strong surface warming leads to deeper boundary layers, which in turn allows for dust and pollutants to reach higher levels where the stronger winds can transport them further away from the source regions^129^. This is seen in the dust AOD trend (Fig. 2d). As a result of the warmer temperatures, the SHL and the AHL have shifted polewards (Fig. 2b) in line with a widening of the Hadley Cell. An analysis of the respective indices revealed that the SHL has been moving eastwards, at a rate of about 0.01° year^−1^, and the AHL northwestwards, at approximately 0.028° year^−1^ (Supplementary Fig. 1b,c), suggesting they have been getting closer to each other, and both have also been strengthening at a rate of 0.1–0.2 m year^−1^ (Supplementary Fig. 1a) in the last four decades. While the SHL exhibits considerable intra-seasonal variability (as the AHL), the change in its intensity is largely controlled by the variations in the background temperature^130^.

The intertropical discontinuity region (ITD) over West Africa^131^ and the Arabian Peninsula^116^ has also been migrating polewards at 0.01–0.02° year^−1^ (Supplementary Fig. 1d), in line with the northward shift of the thermal lows. The subtropical highs have also been shifting, with the one over North Africa migrating westwards by roughly 0.02° year^−1^ (not shown). These changes in the location of the subtropical highs and thermal lows have contributed to an increased dust loading over eastern Africa and the western Arabian Peninsula, with a hotspot around the southern Red Sea and the Gulf of Aden (Fig. 2d). Dust can accumulate here due to the convergence of the southwesterly monsoon winds over the Arabian Sea with the northwesterly Shamal winds from the Arabian Peninsula^132^, and with a contribution from dust advected from northeastern Africa by an enhanced Tokar Gap westerly jet^133^. The steep topography of the region also helps to locally confine the aerosols. The intensification of the Shamal winds^123^, which arises from a strengthening of the AHL and increased pressure gradient between the subtropical high and the thermal low, leads to enhanced dust emissions in the Arabian Peninsula (cf. Figure 2b,d).

In the MENA region, dust loading has been increasing in the summer, with the dust optical depth going up at a rate of 0.0006 year^−1^. Together with the increase in cloud cover (0.037% year^−1^), precipitable water (0.635 kg m^−2^ year^−1^) and precipitation (4.4 × 10^−8^ mm h^−1^ year^−1^), this translates into a reduction of the surface downward shortwave and increase of the downward longwave radiation fluxes by ~ 0.3 W m^−2^ year^−1^, respectively (Table 3). The higher moisture content over the Arabian Peninsula and northern Arabian Sea in the summer (Fig. 1a) may arise from the positive trend in the SSTs, as noted by^134^, and consequently enhanced surface evaporation. The increasing temperatures (Fig. 2a) are also capable of holding larger amounts of moisture. It is interesting to note that the marked increase in temperature over Europe is in contrast with a slight cooling trend over parts of Southern Asia such as northern India. In addition to rainfall changes (Fig. 2c), a contributing factor to this is a reduction in aerosol loading over Europe and an increase over South and East Asia (Fig. 2d)^135^. As a result, the meridional temperature gradient at lower levels has been decreasing, yielding a reduced vertical shear of the zonal wind through thermal wind balance, and ultimately leading to a weakening of the upper-level westerly jet.

The CLARA dataset provides further cloud products such as daytime and nighttime, and high, middle and low cloud cover as well as cloud top temperature. The respective intercept and trends are given in Supplementary Figs. 2, 3. Over Africa, the increase in cloud cover is seen mostly during daytime (cf. Supplementary Fig. 2a,b), with typical magnitudes of 0.1% year^−1^. This is expected, and arises from a poleward shift in the ITCZ (Figs. 1a,c,d and 2c). Here, the clouds have been getting deeper, with the cloud top temperature dropping at a rate of up to 0.3 K year^−1^ (Supplementary Fig. 3d), primarily due to an increase in high-level clouds (Supplementary Fig. 3a–c), an indication of deeper convection. Mid-top clouds are the most frequent cloud type in western Sahara in the summer, with a maximum frequency in the evening, and result from the convergence of mass and humidity in the lower Saharan atmospheric boundary layer (SABL) due to the strong surface heating, and divergence in the upper SABL^136^.

Over the Sea of Oman, the increase in cloud cover is almost exclusively during daytime (Supplementary Fig. 2a) at a rate of 0.1% year^−1^, and due to low-level clouds (Supplementary Fig. 3a). Here, morning low-level clouds in the summer are a regular occurrence, and arise from the interaction of the moist air mass from the Arabian Sea with the mountainous terrain over northern Oman and eastern United Arab Emirates^137^. The weakening of the thermal low over India/Pakistan, with a trend of up to 0.025 hPa year^−1^, owing to increased precipitation (0.002 mm h^−1^ year^−1^), and the strengthening of the AHL (0.136 m year^−1^), driven by the strong surface warming (Fig. 2a-c), further encourages the moist air flow from the Arabian Sea into the Sea of Oman, which in turn promotes the development of low-level clouds in the region. Over the Al Hajar mountains in northern Oman and the Sarawat mountains in western Saudi Arabia, convection in the warmer months typically takes place during daytime when the topography-driven flow interacts with the sea-breeze circulation^59,138,139^. Here, there appears to be a shift in the timing of convection from daytime to nighttime (Supplementary Fig. 2a,b). Higher amounts of moisture (0.75 kg m^−2^ year^−1^), clouds (0.25% year^−1^) and dust (0.003 year^−1^ increase in dust optical depth) translate into reduced surface downward shortwave and increased downward longwave radiation fluxes by about 0.3 W m^−2^ year^−1^ (Fig. 1c,d). A comparison of Fig. 1c,d with Figs. 1a,b, 2c,d indicates that clouds have the largest impact, in particular deep tropical convective clouds.

In the colder months, increased precipitable water levels are seen over the Arabian Peninsula, eastern Africa and India at a rate of about 0.6 kg m^−2^ year^−1^, with a drying tendency over central northern Africa at half that rate (Fig. 1a). A comparison with the precipitation plot (Fig. 2c) reveals an eastward shift in the convective regions over equatorial and southern tropical Africa, with the area around the Democratic Republic of Congo experiencing drier (Fig. 2c; − 0.002 mm h^−1^ year^−1^) and warmer (Fig. 2a; 0.03 K year^−1^) conditions and associated lower pressures (Fig. 2b; − 0.01 hPa year^−1^). On the other hand, further southeast around Tanzania there has been a tendency for wetter (0.001 mm h^−1^ year^−1^) conditions in the last four decades.

Owing to lower pressures over equatorial Africa and higher pressures to the south (Fig. 2b), there is increased dust loading over the Gulf of Guinea (Fig. 2d), corresponding to an equatorward shift of the dusty regions with respect to the background state. Cloud cover has been increasing over subtropical northern Africa and the northwestern and southeastern Arabian Peninsula at a typical rate of ~ 0.25% year^−1^ (Fig. 1b). These are mostly low-level clouds (Supplementary Fig. 3a–c) that have been getting shallower (Supplementary Fig. 3d; increase in cloud top temperature by 0.25 K year^−1^), occurring during daytime and nighttime (Supplementary Fig. 2a,b), indicating they are not driven by the local diurnal cycle. Their radiative signature is mostly in the long-wave in the Arabian Peninsula (+ 0.25 W m^−2^ year^−1^), while over Mauritania and Algeria the increased cloud cover cuts the surface downward shortwave (− 0.25 W m^−2^ year^−1^) and enhances the longwave (+ 0.5 W m^−2^ year^−1^) radiation flux (cf. Figure 1c with d). Although they are not associated with increased precipitation (Fig. 2c), these clouds are probably driven by moisture plumes originating in the tropical Atlantic and equatorial Africa (Fig. 1a). Such excursions of tropical moisture into the higher latitudes can take the form of atmospheric rivers^140^, which are becoming more frequent in the region^56,141^, a trend which is likely to continue in a future warming climate^142^.

Over land areas the near-surface warming is more pronounced in JJA, while over the oceans the air temperature trend is roughly the same in both summer and winter seasons (Fig. 2a). Focusing on the MENA region, the increase in precipitable water, air temperature and dust loading seen in the summer season are also present in the colder months, although a negative trend in the cloud cover (− 0.03% year^−1^) and precipitation rate (− 2.1 × 10^−6^ mm h^−1^ year^−1^) yield an increase (0.17 W m^−2^ year^−1^) in the downward shortwave radiation flux at the surface as opposed to the decrease (− 0.33 W m^−2^ year^−1^) seen in the warmer months (Table 3).

### Performance and Trends of CMIP6 Multi-Model Ensemble

**Figure 3:**
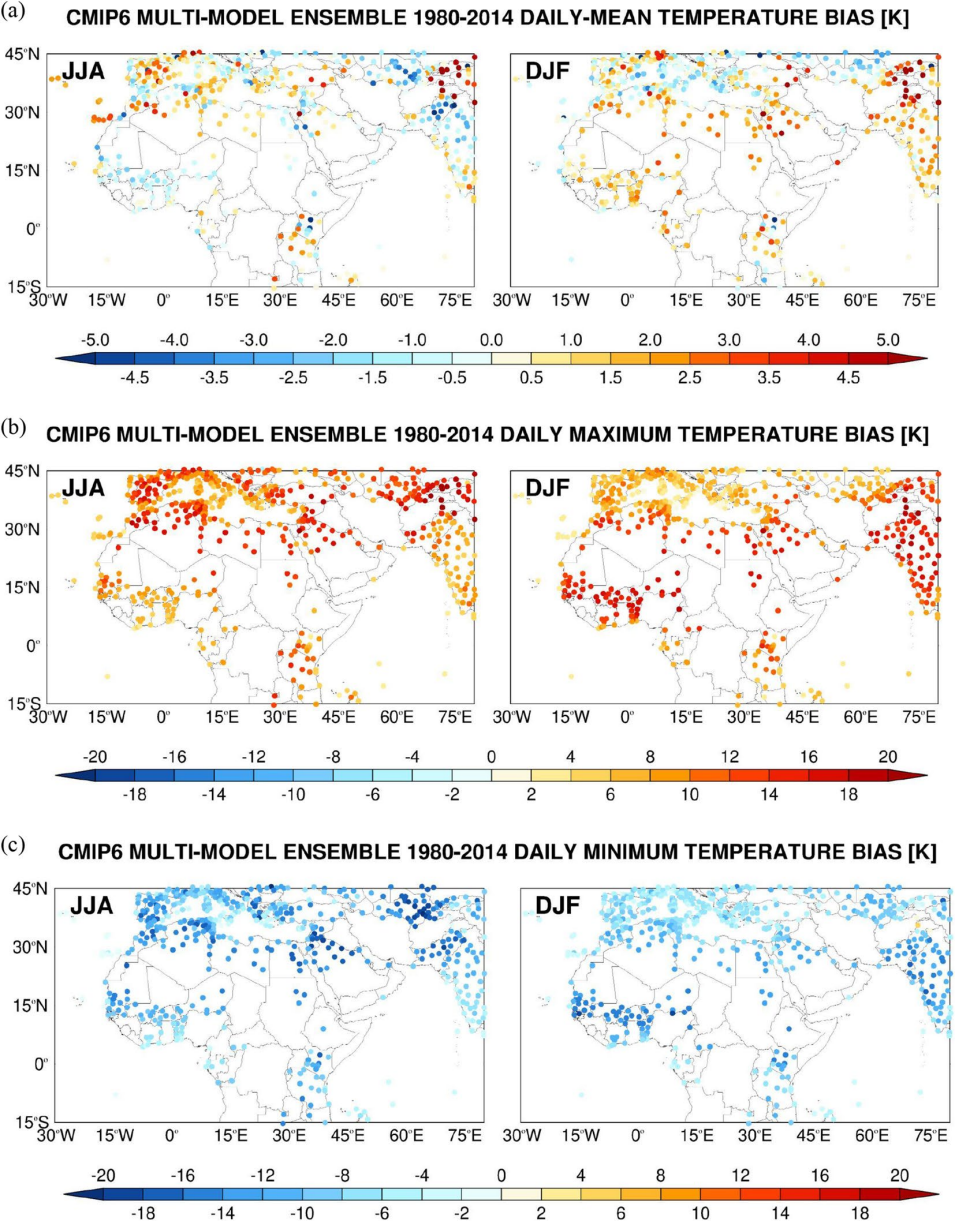
Spatial Evaluation of CMIP6 Multi-Model Ensemble (MME) Air Temperature Simulations against Station Observations. CMIP6 MME Mean (MMEM) bias in the daily (**a**) mean, (**b**) maximum and (**c**) minimum air temperature (K) averaged over 1980–2014 at the location of 574 NOAA GSOD stations. The skill scores are shown for the boreal summer (JJA) in the left plot and for the boreal winter (DJF) in the right plot.

**Figure 4:**
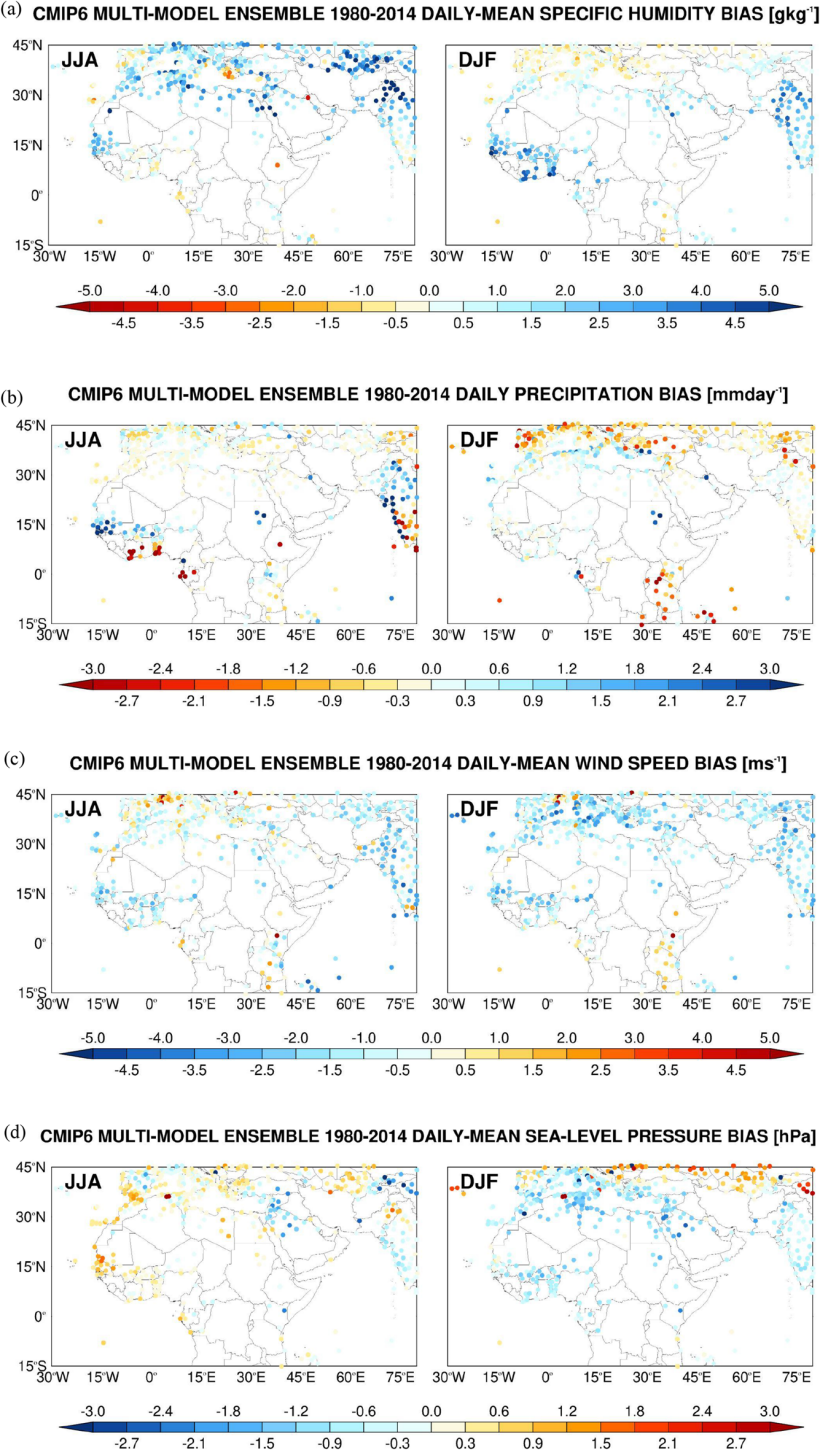
Spatial Evaluation of CMIP6 Multi-Model Ensemble (MME) Humidity, Temperature, Wind Speed and Precipitation Simulations against Station Observations: CMIP6 MME Mean (MMEM) bias in the (**a**) daily-mean specific humidity (g kg^−1^), (**b**) daily precipitation (mm day^−1^), and daily-mean (**c**) 10-m wind speed (m s^−1^) and (**d**) sea-level pressure (hPa) averaged over 1980–2014 at the location of 574 NOAA GSOD stations. The skill scores are shown for the boreal summer (JJA) in the left plot and for the boreal winter (DJF) in the right plot.

**Figure 5:**
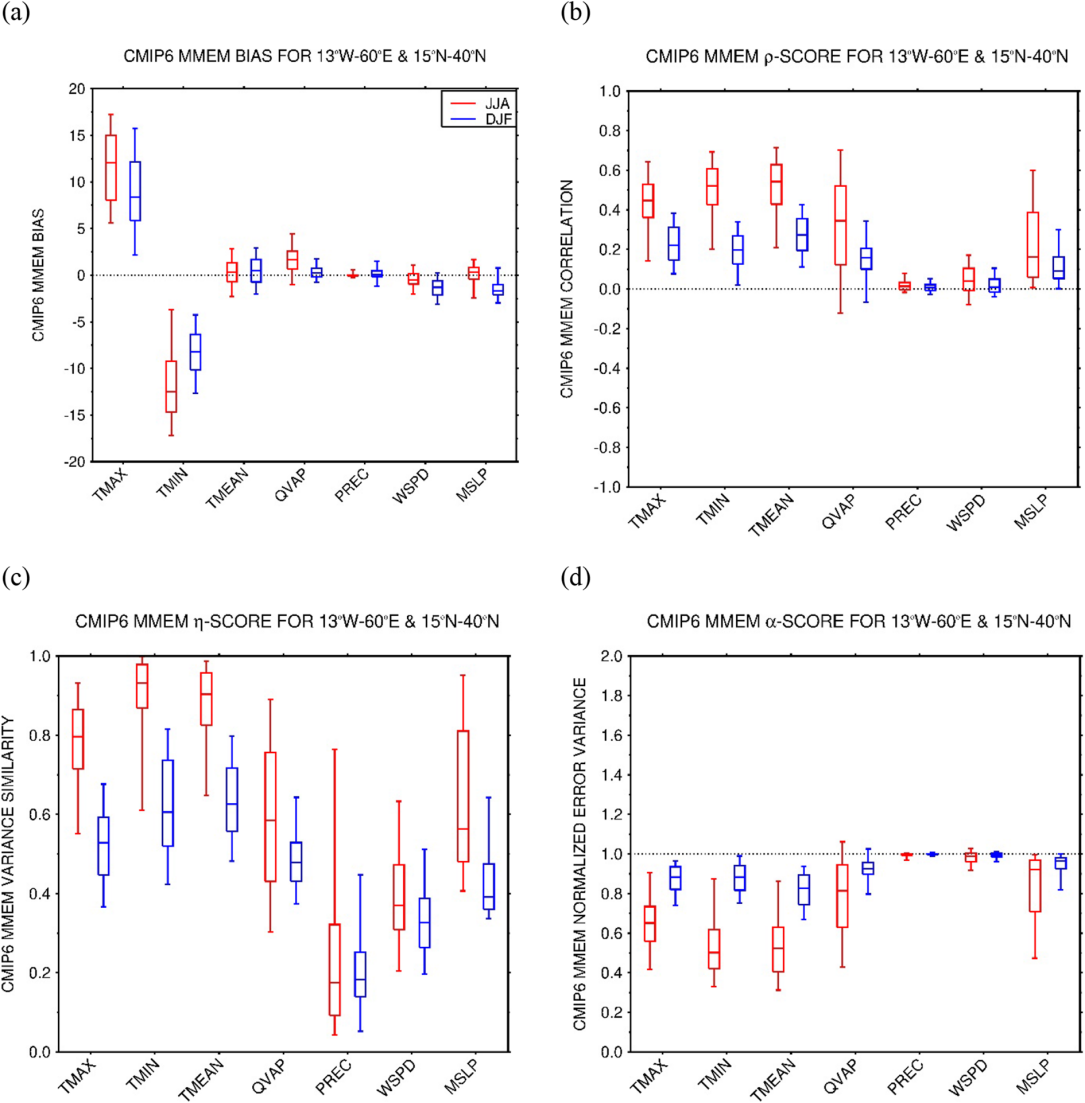
Statistical assessment of CMIP6 MME simulations against station observations: CMIP6 MME (**a**) bias, (**b**) correlation (ρ), (**c**) variance similarity (η) and (**d**) normalized error variance (α) for the daily maximum (TMAX), minimum (TMIN) and mean (TMEAN) air temperatures (bias in K), daily-mean specific humidity (QVAP; g kg^−1^), daily precipitation (PREC; mm day^−1^), and daily-mean 10-m wind speed (WSPD; m s^−1^) and sea-level pressure (MSLP; hPa) averaged over 1980–2014 at the location of 206 NOAA GSOD stations in the MENA region (13° W–60° E, 15°–40° N). The scores are given for the boreal summer (JJA; red) and winter (DJF; blue) seasons. The verification diagnostics are computed as follows: at the location of each station and for a given season, the skill scores are extracted using all the days in the historical period. The box plots display the scores for the summer and winter seasons, giving the 5th, 25th, 50th (median), 75th and 95th percentiles. The correspondent spatial scores are given in Supplementary Fig. 6.

The performance of the CMIP6 MME constructed from the models listed in Table 1 is evaluated against the NOAA GSOD data. Figures 3, 4 shows the summertime and wintertime bias in the daily maximum, minimum and mean air temperature, daily-mean specific humidity and wind speed, and daily precipitation, with the skill scores for the MENA region given in Fig. 5 (temporal scores) and Supplementary Fig. 6 (spatial scores). The CMIP6 MME inter-model spread is presented in Supplementary Figs. 4, 5. The CMIP6 MMEM captures relatively well the observed daily-mean air temperature with biases generally within ± 2 K (Fig. 3a), even though this largely comes from a cancellation of an overestimation of the daily maximum with an underestimation of the daily minimum temperatures (Fig. 3b,c) in particular in drier regions and seasons, as also noted by^143^.

Numerical models generally exhibit larger temperature biases in arid and semi-arid regions that are more pronounced in the warmer months and at night^116,144–146^ which are only slightly alleviated by updating the surface properties^147–149^. They are likely tied to deficiencies in the physics parameterizations in the land surface and radiation schemes, and an incorrect representation of the concentration of aerosols, greenhouse gasses and soil moisture^43,59,150–154^. This is reflected in the bias statistics for the MENA region (Fig. 5a) with poorer scores in the summer season and magnitudes in the range 10–15 K for the maximum and minimum temperatures. The day-to-day variability of air temperature in the region in winter months is largely controlled by the passage of mid-latitude weather systems ^122^, the timing of which will not be fully captured by the CMIP6 MMEM, and hence the lower correlations of ~ 0.2 (Fig. 5b). In the summer, mesoscale circulations such as the land-sea breeze play an important role in the day-to-day temperature fluctuations^122^, which are better captured by the CMIP6 MMEM, yielding higher ρ scores of 0.4–0.6 (Fig. 5b). For the temperature simulations, in general ρ/η < 1, indicating that phase errors prevail over amplitude errors (Fig. 5c). This suggests that more effort should be placed in getting the timing of the passage of baroclinic weather systems and land-sea breeze occurrence right than in correctly predicting their intensity. The normalized error variance (α) score (Fig. 5d) is less than 1, which stresses the CMIP6 MMEM temperature simulations can be regarded as useful, being more skillful than a random simulation. Spatial scores are slightly higher, in particular for η and consequently α (cf. Figure 5 with Supplementary Fig. 6), indicating the CMIP6 MMEM has a higher skill in capturing the spatial strength of the temperature fluctuations, more linked to the land-sea mask, than the intensity of its temporal variability. A close inspection of Fig. 3 reveals the biases are larger over the high-terrain, in particular over the Himalayas, Atlas Mountains, Alps and the higher elevations of Spain and Turkey. Here, the coarser spatial resolution of the CMIP6 MMEM (2° × 1.5°) does not allow for a proper representation of the observed topography, as also evidenced by the higher uncertainty values (Supplementary Fig. 4).

The near-surface specific humidity biases (Fig. 4a) are generally small, typically of 1–2 g kg^−1^, with positive values in the summer mid-latitudes, consistent with the higher temperatures (Fig. 3) and associated increased surface evaporation, as well as in the monsoon region of South Asia where it is driven by a stronger moisture transport^155^. It is interesting to note that over West Africa in the summer the CMIP6 MMEM simulates drier and less moist conditions along the Gulf of Guinea coastline but higher rainfall totals and specific humidity values further north (Fig. 4a,b). This reflects an incorrect representation of the West African monsoon, as noted e.g. by ^156^, and in line with the larger uncertainty (Supplementary Fig. 5a,b). Here, in winter, the higher air temperatures (Fig. 3a), which come from a larger overestimation of the daily maximum temperatures (Fig. 3b), promote the advection of the more moist air from the Gulf of Guinea inland, which explains the larger humidity values (Fig. 4a).

The tendency of the CMIP6 MMEM to overestimate the specific humidity in the MENA region is highlighted in Fig. 5a, with slightly poorer scores in the warmer months. A possible explanation is that the higher daily maximum temperatures (Fig. 3b) lead to excessive evaporation from the adjacent water bodies, in particular the Mediterranean Sea, Red Sea, Arabian Gulf and Arabian Sea, which increases the humidity levels. This is consistent with the fact that the largest biases are generally seen in coastal and near-coastal sites, with the magnitude of the biases decreasing away from the shore (Fig. 4a). The ρ, η and α scores are largely comparable to those of the daily-mean temperature (Fig. 5b,d and Supplementary Fig. 6b,d), even though η is typically lower, and consequently α is higher. This is likely a reflection of a deficient simulation of the intensity of the moisture advection by the land-sea breeze in the summer, probably arising from an incorrect representation of the land-sea temperature gradient, and of the baroclinic weather systems in the colder months, suggesting a mismatch in the position of the storm track. In fact^157^, found that the mid-latitude storm track in the North Atlantic Ocean is slightly displaced further south over Europe in CMIP6 models in winter compared to the ERA-5 reanalysis^76^. This is consistent with the humidity (Fig. 4a) and precipitation (Fig. 4b) biases in the region, with the CMIP6 MMEM tending to over-predict the precipitation in humid regions and under-predict in arid and semi-arid regions (cf. Figure 4a).

Given the meager and irregular amounts of precipitation that fall in the MENA region^158^, and the difficulty of numerical models, in particular coarse-resolution models, in simulating it^159^, it is not surprising that the skill scores are low for this field (Fig. 5 and Supplementary Fig. 6). The correlations are typically ~ 0–0.2 (Fig. 5b) indicating the CMIP6 MMEM does not capture well the timing and spatial extent of these events, most likely because of an incorrect representation of the mid-latitude baroclinic systems in the colder months, as noted before and seen in the inter-model spread (Supplementary Fig. 5b), and the failure to replicate the necessary ingredients for summertime convection and its spatial coverage ^137,151,160^. The normalized error variance is mostly in the range 0.8–1, Fig. 5d and Supplementary Fig. 6d, confirming the CMIP6 MMEM simulations are only marginally useful, due to both phase (Fig. 5b and Supplementary Fig. 6b) and amplitude (Fig. 5c and Supplementary Fig. 6c) errors.

As far as the near-surface wind speed is concerned, there is generally a slight underestimation by about 0.5–1 ms^−1^, with an overestimation over the high terrain in particular over East Africa and southern Europe (Fig. 4c). This is attributed to an incorrect representation of the topography and land-sea mask. Despite the smaller biases (Fig. 5a), the day-to-day variability of this field is poorly simulated, with correlation values around zero (Fig. 5b), worse than those for the other variables shown except for precipitation. This is not surprising, as the wind field exhibits a higher spatial and temporal heterogeneity compared to temperature and humidity, which is also reflected in the uncertainty maps (cf. Supplementary Fig. 4 with Supplementary Fig. 5b). The CMIP6 MMEM wind simulations, and as is the case for the precipitation, are only marginally useful in the MENA region, with α scores around 1 (Fig. 5d). Spatially, the scores are slightly higher (cf. Figure 5 with Supplementary Fig. 6), as spatial patterns, which are more controlled by the land-sea mask and topography, are better captured compared to the temporal fluctuations. The last field presented in Figs. 4, 5 is the sea-level pressure. Due to a southward shift in the position of the storm track, the biases in the MENA region are mostly negative in the colder months, Figs. 4d and 5a, with a higher inter-model uncertainty here as well (Supplementary Fig. 5d). The lower correlations (ρ ~ 0.2; Fig. 5b) and variance similarities (η ~ 0.5; Fig. 5c) likely reflect deficiencies in the representation of the mid-latitude weather systems in the colder months, and the variability of the subtropical highs^113,114^ and thermal lows^116,161^ in the summer. In any case, α < 1, Fig. 5d, a sign of useful simulations. The spatial extent of the summertime thermal lows and subtropical highs is well captured, as evidenced by ρ ~ 0.9, η ~ 0.95 and α ~ 0.1 (Supplementary Fig. 6b–d), while in the colder months the span of the mid-latitude baroclinic systems is poorly represented with α ~ 0.6–1.

**Figure 6:**
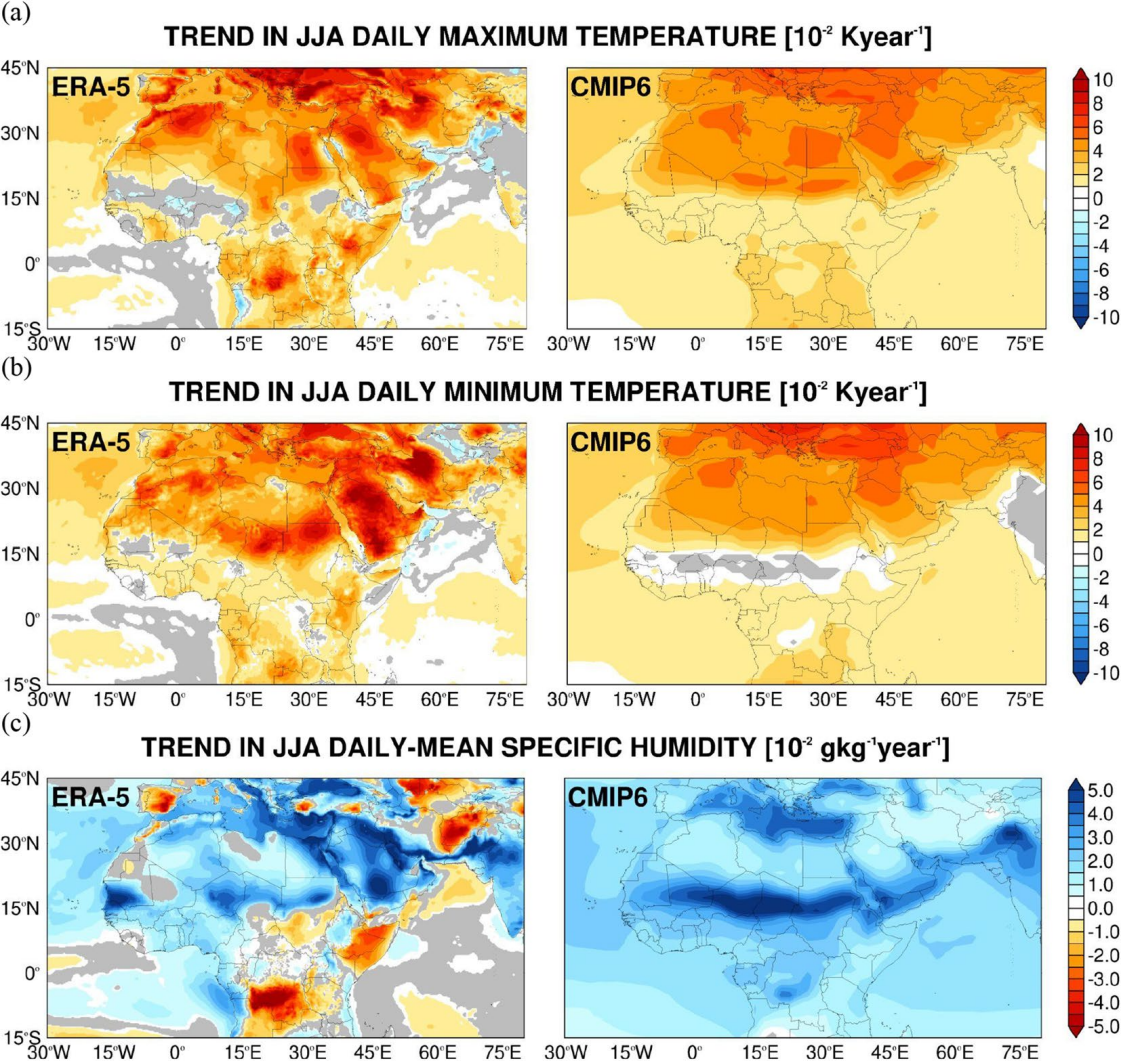
Evaluation of CMIP6 MME air temperature and humidity against ERA-5 reanalysis: (**a**) Linear trend in the seasonal daily maximum air temperature from ERA-5 (left) and the CMIP6 MMEM (right) for the boreal summer season for 1980–2014 (units of 10^−2^ K year^−1^). Regions where the trend is not statistically significant at the 95% confidence level are shaded in grey. The intercept and slope are obtained with the Theil-Sen estimator while the Mann–Kendall test is used for statistical significance. (**b**–**c**) are as (**a**) but for the daily minimum air temperature (10^−2^ K year^−1^) and daily-mean specific humidity (10^−2^ g kg^−1^ year^−1^), respectively. The corresponding plots for the boreal winter season are given in Supplementary Fig. 7.

**Figure 7:**
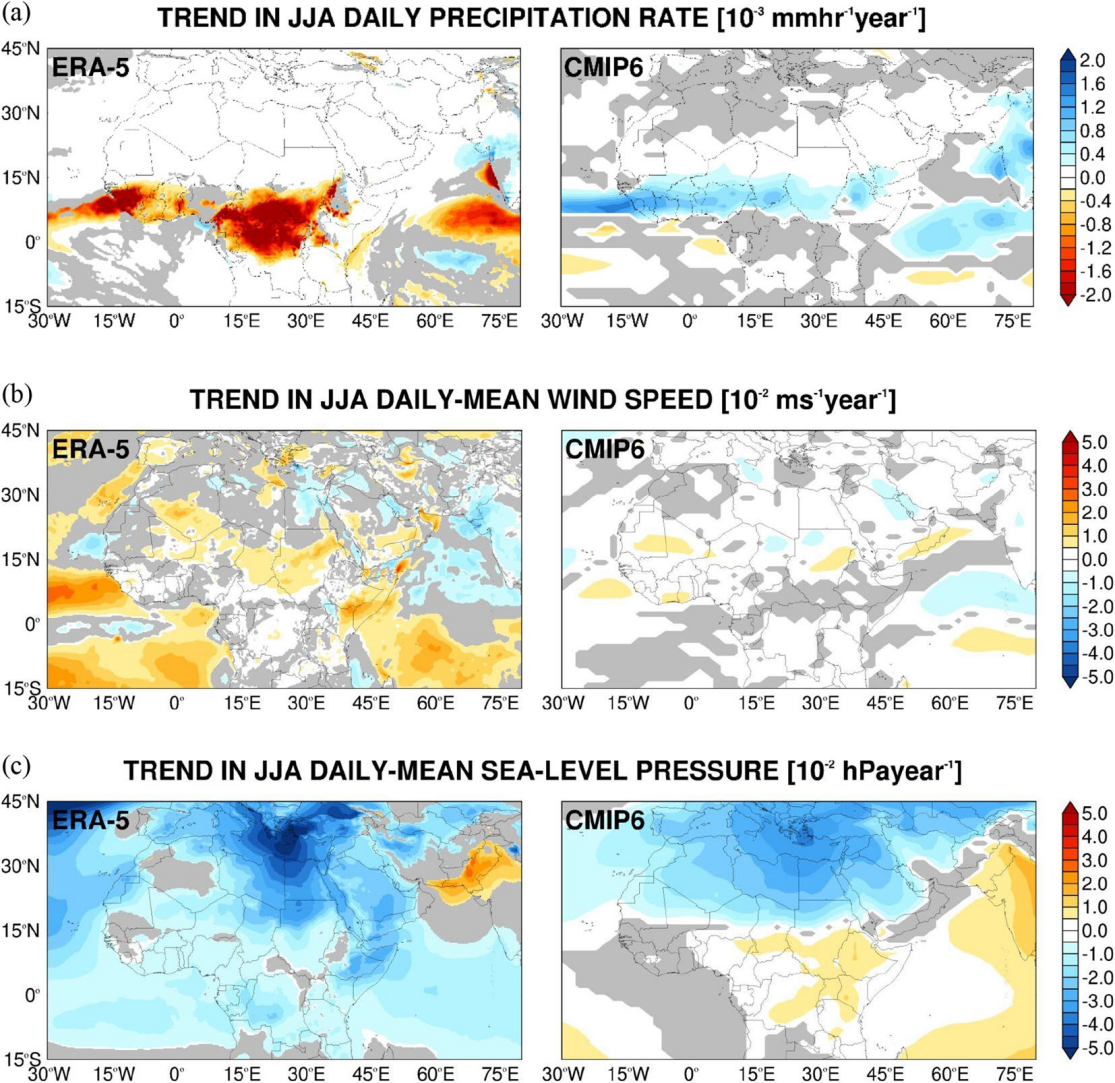
Evaluation of CMIP6 MME Precipitation, Wind Speed and Sea-Level Pressure against ERA-5 Reanalysis: (**a**) Linear trend in the seasonal daily precipitation rate from ERA-5 (left) and the CMIP6 MMEM (right) for the boreal summer season for 1980–2014 (units of 10^−3^ mm h^−1^ year^−1^). Regions where the trend is not statistically significant at the 95% confidence level are shaded in grey. The intercept and slope are obtained with the Theil-Sen estimator while the Mann–Kendall test is used for statistical significance. (**b**–**c**) are as (**a**) but for the daily-mean wind speed (10^−2^ m s^−1^ year^−1^) and sea-level pressure (10^−2^ hPa year^−1^), respectively. The corresponding plots for the boreal winter season are given in Supplementary Fig. 8.

In Figs. 6, 7 and Supplementary Figs. 7, 8, the trend of the daily maximum and minimum air temperatures, daily-mean specific humidity, wind speed, sea-level pressure and precipitation for the CMIP6 MMEM is compared to that of ERA-5 for the historical period of 1980 to 2014. The respective trends in the NOAA GSOD observations are given in Supplementary Figs. 9, 10.

Both the reanalysis, observational data and the model output exhibit a general warming trend in both seasons, with typical amplitudes of 0.05 K year^−1^. In all, the near-surface warming is more pronounced in the Northern Hemisphere, with the more homogeneous spatial patterns in CMIP6 consistent with the fact that an ensemble is used. The peak values over northern Saudi Arabia and parts of southeastern Europe in the summer are captured by the model (Fig. 6a,b). In winter, the warming in the daily maximum and minimum temperatures is more comparable, with the highest trend values over central and eastern Africa and the Middle East, exceeding 0.1 K year^−1^ in parts of western Iran and eastern Iraq (Supplementary Fig. 7a,b). This tendency is also present in the GSOD data (Supplementary Fig. 9), which shows a negative tendency in the daily minimum temperature over parts of southwestern Europe of about − 0.025 K year^−1^ where the ERA-5 trend is not statistically significant. The main difference between the ERA-5/GSOD and the CMIP6 MMEM is over southwest Asia in winter, where the model shows warming of 0.05 K year^−1^ while the reanalysis and observation data give a cooling trend in some parts (Supplementary Figs. 5a,b and 7a,b). The overestimation of the surface solar radiation flux over land in CMIP6 models, which has a larger magnitude over this region in winter and spring^162,163^, may explain the temperature increase here. Fan et al.^164^ also noted a positive temperature bias and a steeper temperature trend with respect to observations over Southwest Asia and a negative bias and weaker temperature trend over the majority of northern Africa, in line with the trends in Fig. 6a,b and Supplementary Fig. 7a,b. In addition, this is the region and season where the CMIP6 MMEM has the highest uncertainty (Supplementary Fig. 4), with typical values of 4–6 K.

The higher spatial resolution of the reanalysis dataset allows for smaller-scale features to be resolved, which include a decrease in the daily maximum air temperature over the Sea of Oman in the summer of 0.01 K year^−1^ (Fig. 6a), in line with the increased daytime cloud cover in the recent decades (Fig. 1b; Supplementary Fig. 2a), and some cooling patches over southwest Asia in winter (Supplementary Fig. 7 a,b), owing to reduced cloud cover (Fig. 1b) and higher pressures (Fig. 2b).

It is interesting to note that over the Arabian Peninsula and northeastern Africa the highest trends are seen in the daily minimum temperature, in particular in the summer months (Fig. 6a,b and Supplementary Fig. 7a,b), with magnitudes in excess of 0.1 K year^−1^ at some sites, while for the daily maximum temperature the trend values are mostly within 0.05 K year^−1^. This tendency is also present in the GSOD data (Supplementary Fig. 9). It can be explained by the increased column water vapour (Fig. 1a) and dust loading (Fig. 2d), and is in line with^43,58^ who reported that dust generally has a larger effect on the nighttime temperatures in the Middle East.

In the CMIP6 MMEM, there is a poleward shift of the ITCZ over Africa in summer and an equatorward shift in winter (Figs. 6c, 7a and Supplementary Figs. 7c and 8a), even though there is considerable variability in the MME (Supplementary Fig. 3d–e). The northward displacement of the ITCZ is in line with the more pronounced warming trend in the Northern Hemisphere (Fig. 6a–b and Supplementary Fig. 7a–b), with the humidity exhibiting a similar tendency (Fig. 6c and Supplementary Fig. 7c). Some of these tendencies, in particular for the precipitation and over tropical Africa, are not present in ERA-5, with the drying over the Iberian Peninsula in the summer of up to 0.05 g kg^−1^ year^−1^ seen in the reanalysis data absent in the CMIP6 MMEM (Fig. 6c), which predicts a general increase in near-surface humidity at a rate of 0.01–0.04 g kg^−1^ year^−1^ in both seasons throughout the whole region (Fig. 6c and Supplementary Fig. 7c). The precipitation trends in GSOD are mostly not statistically significant (Supplementary Fig. 10b). As far the specific humidity tendencies are concerned, most are also seen in ERA-5, such as the drying over the Iberian Peninsula and parts of Southwest Asia in the summer months, and the general moistening around the Mediterranean Sea and neighboring regions, which is more pronounced in the warmer months. The drying tendencies over northeastern Africa in both seasons, extending to the southeastern Arabian Peninsula in the colder months present in ERA-5, are not seen in the observational data.

Over West Africa, the CMIP6 MMEM simulates an increase in rainfall during 1980–2014 by about 0.001 mm h^−1^ year^−1^ while in ERA-5 there is a slight drying tendency at roughly twice that magnitude (Fig. 7a and Supplementary Fig. 8a). Gbode et al.^165^ reported that ERA-5 predicts excessive drying in the region compared to observational datasets, even though the GSOD trend is not statistically significant here (Supplementary Fig. 10a). The drying trend over equatorial Africa in ERA-5 is in contrast with the CMIP6 MMEM’s wetter signal (Figs. 6c and 7a and Supplementary Figs. 7c and 8a), with the latter exhibiting higher positive precipitation trends in the Southern Hemisphere mostly in winter. This can be attributed to the warming tendency in the CMIP6 MMEM (Fig. 6a,b and Supplementary Fig. 7a,b), consistent with a stronger Hadley Cell. In any case, the substantial uncertainty in the CMIP6 MME precipitation data over this region (Supplementary Fig. 5b) indicates little agreement between the models and realizations, in particular with respect to the position of the ITCZ and the storm track region in the Northern Hemisphere winter. The wind speed tendency in the reanalysis dataset (Fig. 7b and Supplementary Fig. 8b) shows a stronger Asian summer monsoon low-level flow over the Arabian Sea and adjacent regions by 0.01–0.02 m s^−1^ year^−1^, except over coastal northwestern India, in line with the increased precipitation here (Fig. 7a and Supplementary Fig. 8a). This tendency is partially present in the CMIP6 MMEM, with the weakening of the near-surface wind speed over India and neighboring regions also seen in the GSOD data (Supplementary Fig. 10c).

The Asian winter monsoon circulation has also become more intense during 1980–2014, as does the West African monsoon flow by 0.01–0.04 m s^−1^ year^−1^. This is consistent with the temperature (Fig. 6a,b and Supplementary Fig. 7a,b) and specific humidity (Fig. 6c and Supplementary Fig. 7c) trends seen in the Arabian Sea and neighboring areas. The CMIP6 MME fails to predict this strengthening of the flow, most likely because of the warming tendency over southwest Asia not seen in the reanalysis data (Supplementary Fig. 7a,b), which weakens the land-sea pressure gradient and therefore the monsoon circulation .

The sea-level pressure trends largely match those of air temperature, with the warming tendency leading to lower pressures and vice-versa (cf. Figures 6a,b, 7c; Supplementary Figs. 7a,b, 8c). For this field there is a general agreement between the CMIP6 MMEM, ERA-5 and GSOD, with the most notable difference being over the central and eastern Mediterranean in winter, where a decrease is seen in the first two by 0.01–0.04 hPa year^−1^ while in GSOD a positive tendency of a magnitude twice as large is present at the vast majority of the stations. A possible reason is a shift in the position of the mid-latitude storm track, which has become wavier (i.e. higher sinuosity) in recent decades^119,166^. Changes in the polar jet may explain the increase in wind speed in central parts of the Mediterranean owing to steeper pressure gradients (Supplementary Fig. 10d), a trend not present in both ERA-5 and CMIP6 MMEM (Fig. 7c and Supplementary Fig. 8c).

Despite the aforementioned discrepancies, and accounting for the uncertainty (Supplementary Figs. 4, 5), the CMIP6 MMEM generally simulates the trends seen in the reanalysis and observational data over the historical period. This, together with its good performance when compared to in-situ data shown in Figs. 3, 4, and 5, gives confidence in using the CMIP6 MMEM to explore future climate change projections in the extended MENA region.

### Projected changes based on CMIP6 multi-model ensemble

Some aspects of future climate change for the period 2066–2100 and for the more extreme SSP5-8.5 scenario are investigated using the projections of the CMIP6 MME. The changes in the duration of the seasons for the Middle East and North Africa based on the percentile technique proposed by^110^ applied to the daily-mean air temperature are explored. The lack of precipitation in these areas precludes using precipitation-based methods as noted in^167^. The seasons in the Middle East and North Africa are projected to change by up to two days in 2066–2100 compared to 1980–2014, with a longer spring and a shorter autumn, with only the former and for the Arabian Peninsula being statistically significant at the 95% confidence level (not shown). This suggests that the warming is likely to be felt roughly equally throughout the year (i.e. the baseline is projected to increase but the annual variability will stay about the same as in the present climate).

**Figure 8:**
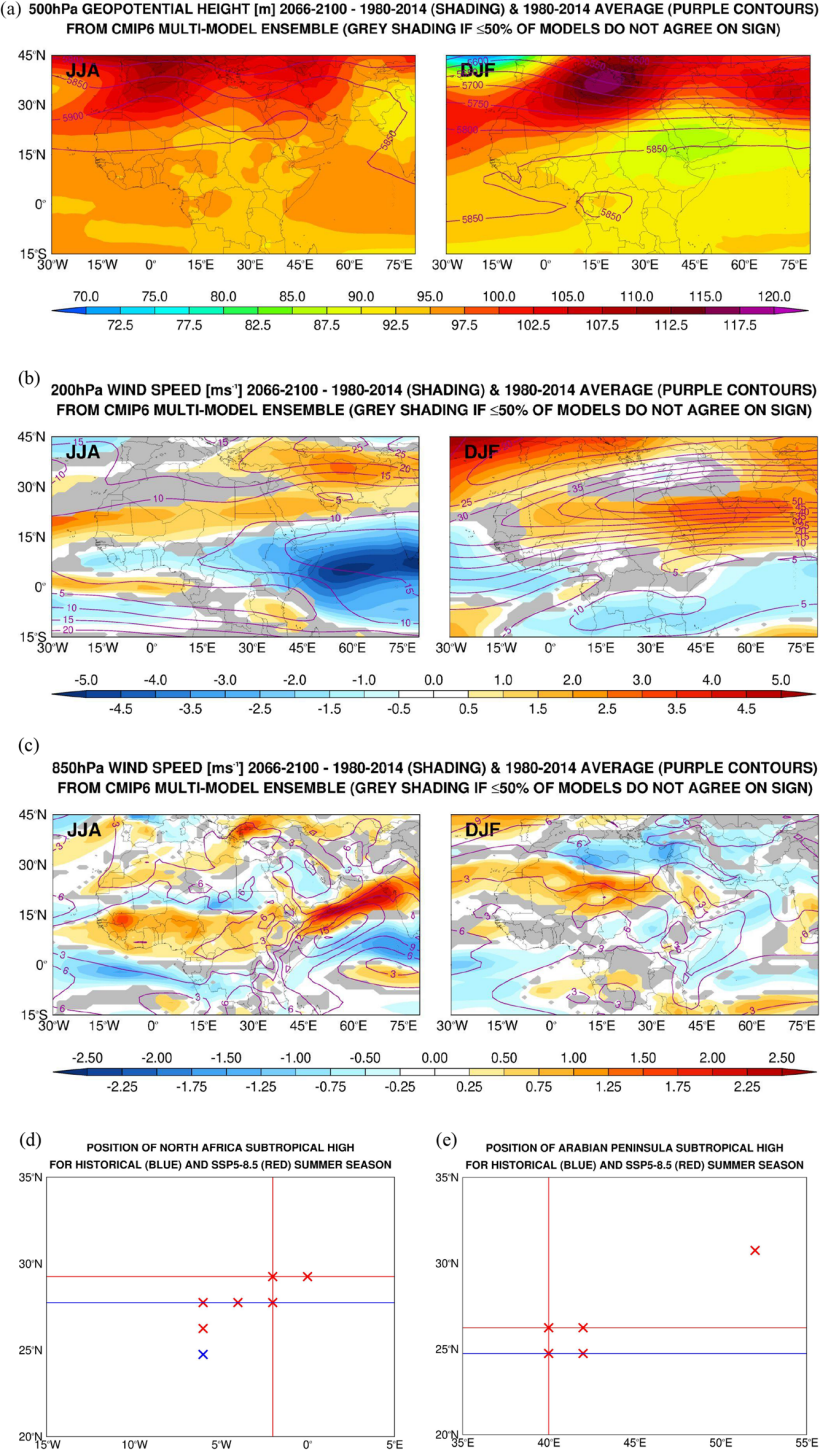
Projected Changes in CMIP6 MME for SSP5-8.5 Scenario: (**a**) Seasonal-mean 500 hPa geopotential height (m) difference between the climate change (2066–2100) and historical (1980–2014) periods (shading) and the climatological values (purple contours) averaged over the summer (JJA; left) and winter (DJF; right) seasons from the CMIP6 MME. Regions where 50% or less of the 10 CMIP6 models considered do not agree on the sign are masked in grey. (**b**–**c**) are as (**a**) but for the 200 hPa and 850 hPa wind speed (m s^−1^), respectively. (**d**) and (**e**) scatterplots of the longitude and latitude of the center of the North African (0°–35° N; 15° W–30° E) and Arabian Peninsula (5°–35° N; 35°–60° E) subtropical anticyclones, respectively, for the summer season of each of the historical (blue; 1980–2014) and climate change (red; 2066–2100) years. The vertical and horizontal lines indicate the median value of the longitude and latitude, respectively, of the subtropical highs for each period. In both plots, some of the crosses, and the vertical lines, overlap with each other.

The background warming, reflected in the domain-wise increase of 500 hPa geopotential height (Fig. 8a), is projected to have a profound impact on large-scale atmospheric circulation. Figures 8b-c show the historical (1980–2014) and the difference between the future climate (2066–2100) and historical 200 hPa and 850 hPa wind speed, respectively. The corresponding uncertainty maps are given in Supplementary Fig. 11. It is interesting to note that the standard deviation of the upper- and low-level winds in the summer and winter seasons are largely the same in the historical and climate change periods, indicating a similar inter-model variability in the future climate, compared to that in the historical period.

The most notable projected changes at upper-levels are an intensification of the subtropical jet in winter with a roughly 10% increase in the wind speed (Fig. 8b), except over the eastern Mediterranean where the storm track is projected to shift polewards (Fig. 8a), and a marked weakening and slight poleward shift of the tropical easterly jet (TEJ) over the Indian Ocean in the summer, with the wind speed dropping by more than a third. Some of the weakening of the TEJ has been attributed to the projected reduction in rainfall over the eastern tropical Indian Ocean, with the associated upper-level convergence and westerly wind forcing weakening the easterly jet^168^, This will also likely contribute to the equatorward shift of the subtropical jet. While there is a relatively low uncertainty with respect to the TEJ in the CMIP6 MME, with the standard deviation up to a third of the mean value in the core of the jet (Supplementary Fig. 11b), there is more spread in the position and strength of the wintertime subtropical jet (Supplementary Fig. 11c), even though in both cases there is a fairly good agreement between the different models (Fig. 8b). Besides the effects of anthropogenic aerosols and the projected increased meridional temperature gradient, which will yield a stronger jet, the strength and position of the wintertime subtropical jet is modulated by the way the West Pacific Warm Pool is represented in the models^169^. This is because the characteristics of the Warm Pool directly impact tropical convection and hence the extratropical circulation. Models with a climatologically smaller West Pacific Warm Pool area tend to expand more in the future, which increases the strength of the subtropical jet^169^. The weaker jet over the eastern Mediterranean is consistent with a poleward shift of the mid-latitude storm track and reduced cyclogenesis here (Fig. 8a).

A strengthening of the westerly flow over Western Europe and a weakening to the north and south in winter is also seen at lower levels (850 hPa), Fig. 8c, with the projected tropical warming, changes in the stratospheric polar vortex and projected warming of the equatorial Pacific playing a role in the position of the eddy-driven jet^170^). There is, however, considerable spread among the models here, as the standard deviation is comparable to the mean values (cf. Figure 8c with Supplementary Fig. 11e), even though the majority of the models agree on the sign of the projected change.

In the summer, the poleward shift of the Somali Jet and the weakening of the equatorial winds over the northern Indian Ocean are in line with a more active South Asian summer monsoon^171,172^. The Tokar Gap Jet around 19° N in the Red Sea is projected to strengthen in a warming climate, continuing a trend seen in the last couple of decades^133^, with little change in the strength of the Turkana Gap Jet over northwestern Kenya. For all these features the standard deviation of the CMIP6 MME is up to a third of the mean values (cf. Figure 8c with Supplementary Fig. 11d–e), indicating there is a reasonable agreement between the models.

The increased low-level wind speed over West Africa in the summer and over the southern tropical Africa in winter (Fig. 8b,c) is consistent with the projected poleward expansion of the West African monsoon in the summer and the Southern African monsoon in winter^173^. The subtropical highs are projected to shift polewards in a future warming climate, as evidenced by a steeper increase in the 500 hPa geopotential height at higher latitudes (Fig. 8a), in line with a broadening of the Hadley Cells. In Fig. 8d,e, the position of the North African and Arabian Peninsula’s subtropical highs for the historical (1980–2014) and climate change (2066–2100) periods are estimated based on the 500 hPa geopotential height maximum in each region. The subtropical high over North Africa in the summer sits at roughly 2°W and exhibits a poleward shift typically of about 1.5° in 2066–2100 compared to 1980–2014, which is statistically significant at the 95% confidence level. This is in line with the projected northward expansion of the West African summer monsoon (Fig. 8c). Its position is also projected to become more variable in longitude (6°W-0° vs. 6°W-2°W) but less variable in latitude (24.75°–27.75° N vs. 27.75°–29.25° N) in the future. The Arabian Peninsula anticyclone is also projected to shift polewards by 1.5°, a trend that is also significant at 95% confidence level. The Arabian Peninsula’s subtropical high is typically over northwestern Saudi Arabia (40° E), but in a warming climate in some years it is projected to shift further east, into western Iran (52° E), which will impact the atmospheric circulation over the region. Except over the eastern Mediterranean, the uncertainty in the summertime 500 hPa geopotential height in the CMIP6 MME is relatively low (Supplementary Fig. 11a), indicating a good agreement between the models over most of the Sahara and Arabian deserts. A change in the position of the subtropical highs will have an important impact on wind patterns in both regions, which are primarily driven by the pressure gradient between the highs and the thermal lows^137^. The poleward shift of the subtropical anticyclones is also consistent with the projected expansion of the tropics in a warming climate, and with the projected changes in the low-level wind flow over North Africa and the Arabian Peninsula (Fig. 8a,b).

## Summary and conclusions

The arid and semi-arid regions over northern Africa and southwest Asia have been expanding in the last several decades with their impacts aggravated by the rapid population growth and they are likely to become even more extreme in a warming climate. In this work, we investigate the changes in the atmospheric circulation and how they are linked to variations in clouds, moisture, dust and radiation, for an extended Middle East and North Africa (MENA) region that comprises northern and equatorial Africa, southern Europe, the Middle East and southwest Asia. This is achieved using a combination of observational and reanalysis datasets for the present climate, and the projections of a multi-model ensemble (MME) constructed using ten models that participated in the sixth phase of the Coupled Model Intercomparison (CMIP6) project for the future climate.

An analysis of observational and reanalysis products over the last four decades revealed that, in the boreal summer, there has been (1) a northward shift of the Intertropical Convergence Zone (ITCZ) over Africa; (2) an eastward shift of the Saharan Heat Low (SHL), at about 0.01° year^−1^; (3) a northwestward shift of the Arabian Heat Low (AHL), at approximately 0.028° year^−1^, with the two thermal lows getting closer to each other and (4) a poleward shift in the position of the Intertropical Discontinuity (ITD) in both regions, at 0.01–0.02° year^−1^. ERA-5 reanalysis data shows a steeper daily minimum compared to the daily maximum temperature increase over northeastern Africa and the Arabian Peninsula, in particular in the summer months, with at some sites the former exceeding 0.1 K year^−1^, while the latter tends to be within 0.05 K year^−1^, a tendency also seen in the in-situ station data. This has been attributed to higher levels of moisture and dust in the atmosphere, and is also consistent with the fact that the SHL and AHL, which are diagnosed from the low-level atmospheric thickness just before sunrise, are getting closer to each other. Higher nighttime temperatures will likely further exacerbate the mugginess in a region where the combination of heat and humidity at times exceeds the threshold for human habitability^53^. The subtropical high over North Africa has been migrating westward at a rate of about 0.02° year^−1^. The referred changes in the thermal lows and subtropical highs have led to increased dust loadings over eastern Africa and the western Arabian Peninsula, with the dust optical depth increasing at a rate of up to 0.005 year^−1^, and a dust hotspot around the Gulf of Aden and southern Red Sea, where the steep topography also helps to spatially confine the dust. Additionally, there is a noticeable shift in the timing of convection from daytime to nighttime over the southern Arabian Peninsula including the Al Hajar mountains in the United Arab Emirates (UAE) and Oman and the Sarawat mountains in Saudi Arabia and Yemen.

In boreal winter, the main changes in the last few decades have been: (1) An eastward shift in the convective regions over equatorial and eastern Africa, with the associated changes in pressure patterns leading to an southward shift of the dusty regions over tropical Africa into the Gulf of Guinea; (2) increased low-level clouds over the subtropical northern Africa and the northwestern and southeastern Arabian Peninsula, at a rate of about 0.25% year^−1^, and (3) increased excursions of tropical moisture over the eastern tropical Atlantic into southwestern Europe, and from East Africa into the Arabian Peninsula.

In order to explore future changes in the climate of the region, the ten CMIP6 models for which daily simulations for the historical period (1980–2014) and projections for the SSP-5.85 climate change scenario (2066–2100) at a nominal resolution of at least 100 km in latitude or longitude and for which all seven target variables (daily-mean air temperature, wind speed, sea-level pressure, specific humidity, precipitation, and daily maximum and minimum temperatures) are available are considered.

The CMIP6 MME future climate projections for 2066–2100 for the SSP5-8.5 scenario are analysed and compared to the 1980–2014 historical period. Spring is projected to last longer and the autumn to be shorter in the Middle East and North Africa typically by 1–2 days, although these changes are only statistically significant at the 95% confidence level in the Arabian Peninsula and for the spring season. This indicates the warming is projected to be felt roughly uniformly throughout the year.

Monthly data from ten CMIP6 models are used to construct a CMIP6 MME to inspect projected changes in pressure-level fields. At 200 hPa, the most prominent differences between the historical and climate change periods are (1) a projected weakening of the tropical easterly jet in summer, with a magnitude decrease by up to a third, partly driven by a reduction in the eastern tropical Indian rainfall, and (2) a projected strengthening of the subtropical jet in winter with a magnitude increase typically by 10%, except over the eastern Mediterranean where the storm track is projected to shift polewards. At 850 hPa, the northward shift of the Somali Jet and of the westerly flow over West Africa, in line with the projected poleward expansion of the South Asian and West African summer monsoons, are the most noticeable signals. The subtropical highs over North Africa and the Arabian Peninsula are projected to migrate polewards by 1.5°, a trend statistically significant at 95% confidence level, in line with the projected expansion of the Hadley Cells.

The findings of this work highlight the changes in climate patterns over the MENA region that have been occurring during the last four decades and summarize the projected changes in the tropospheric circulation for the period 2066–2100. These outcomes are important not only for the scientific community, but also for policymakers, investors and government entities in the framework of the global effort to build climate resilience and foster climate change mitigation initiatives such as energy transition towards renewable sources for which the MENA region can be a key provider.

Finally, our results stress on the need for higher-resolution, optimized modelling products for North Africa, southwest Asia and southern Europe, that compare well with the available observational and reanalysis datasets for the recent past, so as to have confidence in their future climate change projections. It would also be important for such models to explicitly predict the amount of dust and other pollutants in the atmosphere, which are abundantly present in the region^78^, so as to account for the impact of the atmospheric composition on the radiation, atmospheric circulation, convective activities and rainfall. An accurate representation of the spatial and temporal variability of greenhouse gasses is also paramount, as recent studies showed an increasing trend and a heterogeneous spatial pattern in the MENA region^153,154^.

### Supplementary Information

Supplementary Figures.

## Data Availability

The observational and reanalysis products considered in this study are freely available online: (1) the Morphed Integrated Microwave Imagery at the Cooperative Institute for Meteorological Satellite Studies—Total Precipitable Water version 2 (MIMIC-TPW2) data is available at the Cooperative Institute for Meteorological Satellite Studies University of Wisconsin-Madison website ^174^; (2) the Climate Monitoring Satellite Application Facility Cloud, Albedo and Surface Radiation dataset produced from the measurements collected by the Advanced Very High Resolution Radiometer data—second edition (CLARA-A2) data is downloaded from the Copernicus Climate Change Service website ^175–177^; (3) the Clouds and the Earth’s Radiant Energy System (CERES) data is extracted from the National Aeronautic and Space Administration (NASA) Langley Research Center’s website ^178^; (4) the Modern-Era Retrospective analysis for Research and Applications version 2 (MERRA-2) data is obtained from the NASA’s Goddard Earth Sciences Data and Information Services Center (GES DISC) website ^179–181^; (5) the Integrated Multi-Satellite Retrievals (IMERG) for the Global Precipitation Measurement (GPM) mission precipitation rate is also available from NASA’s GES DISC website ^182^; (6) the ERA-5 reanalysis data is accessed on the Copernicus Climate Change Service Climate Data Store website ^183,184^; (7) the Global Surface Summary of the Day (GSOD) data is downloaded from the National Oceanic and Atmospheric Administration National Centers for Environmental Information website ^87^; (8) daily and monthly predictions and projections by numerical models that are part of the sixth phase of the Coupled Model Intercomparison Project (CMIP6) are freely available at the World Climate Research Programme website ^89^. All figures were generated using the Interactive Data Language (IDL; ^185^) software version 8.8.1.

## Code availability

The scripts used to process all observational and modeled datasets can be obtained upon reasonable request from the corresponding author (diana.francis@ku.ac.ae).
