# Supplementary material for: Recent and projected changes in climate patterns in the Middle East and North Africa (MENA) region
Source: Sci Rep. 2024 May 4;14:10279. doi: 10.1038/s41598-024-60976-w (PMC11069548; doi:10.1038/s41598-024-60976-w)
Supplement: Supplementary file 1 — Supplementary Figures. [file 41598_2024_60976_MOESM1_ESM.docx]

# **Supplementary Information**

| (a) | (b) |
| --- | --- |
| 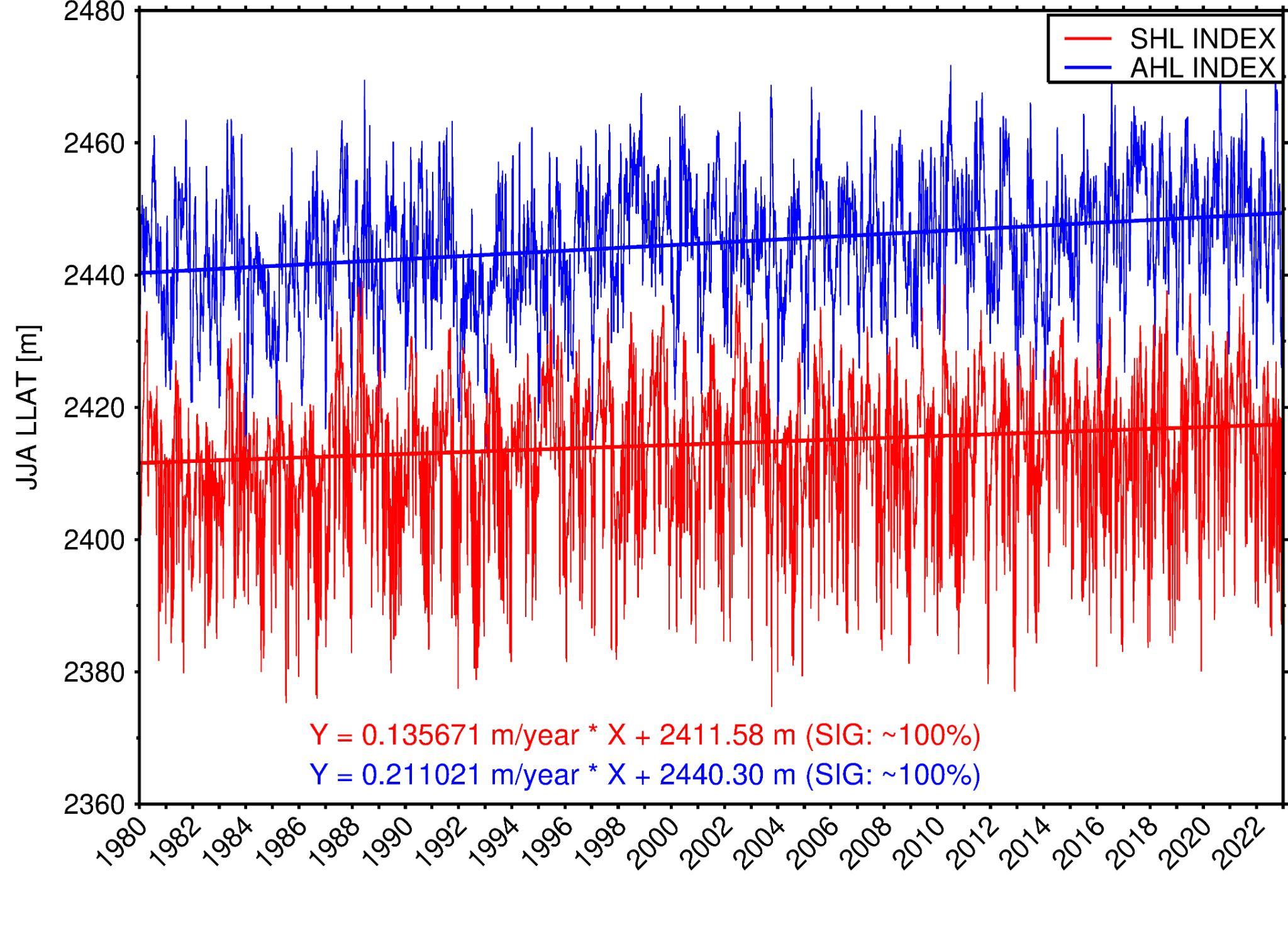 | 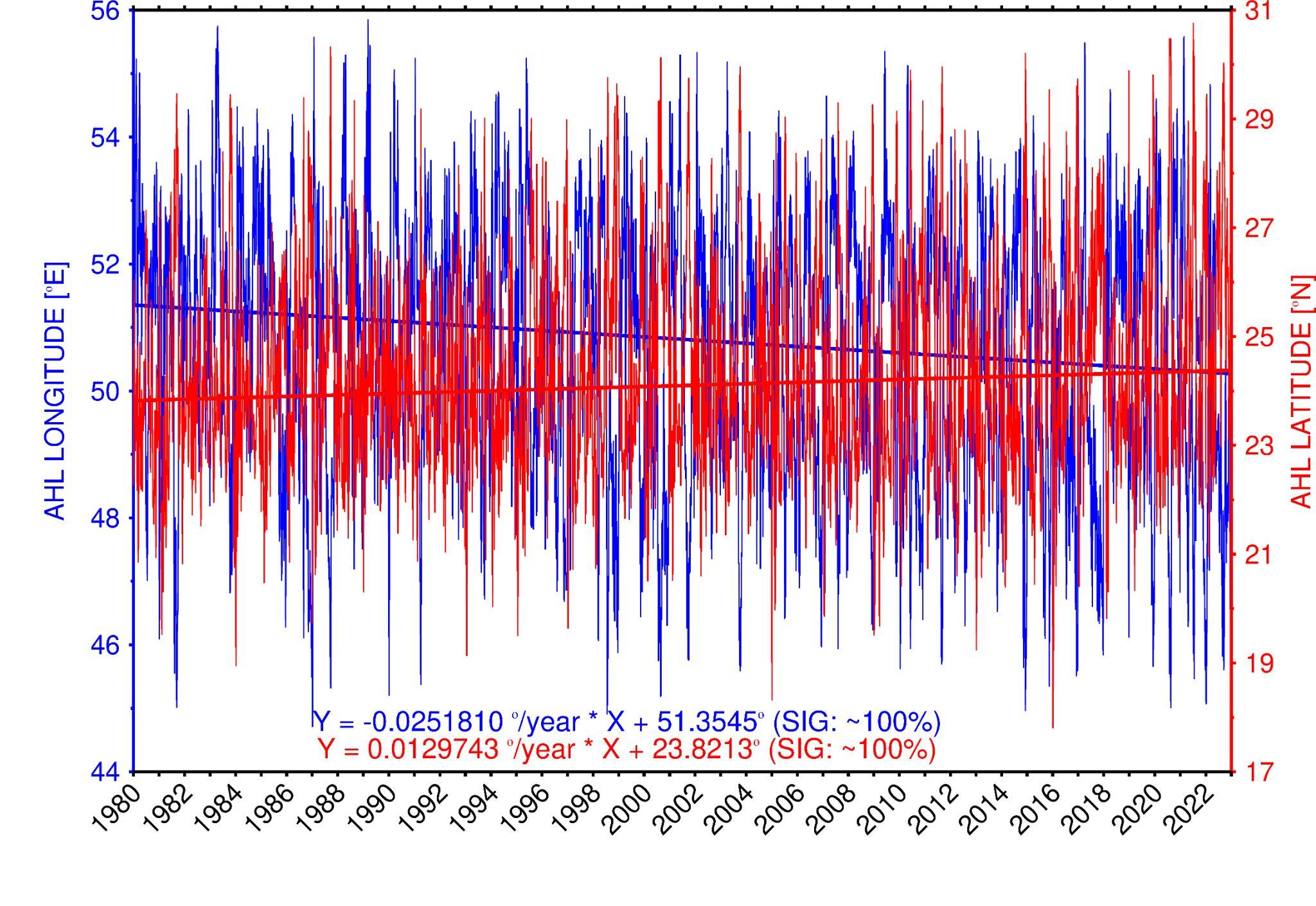 |
| (c) | (d) |
| 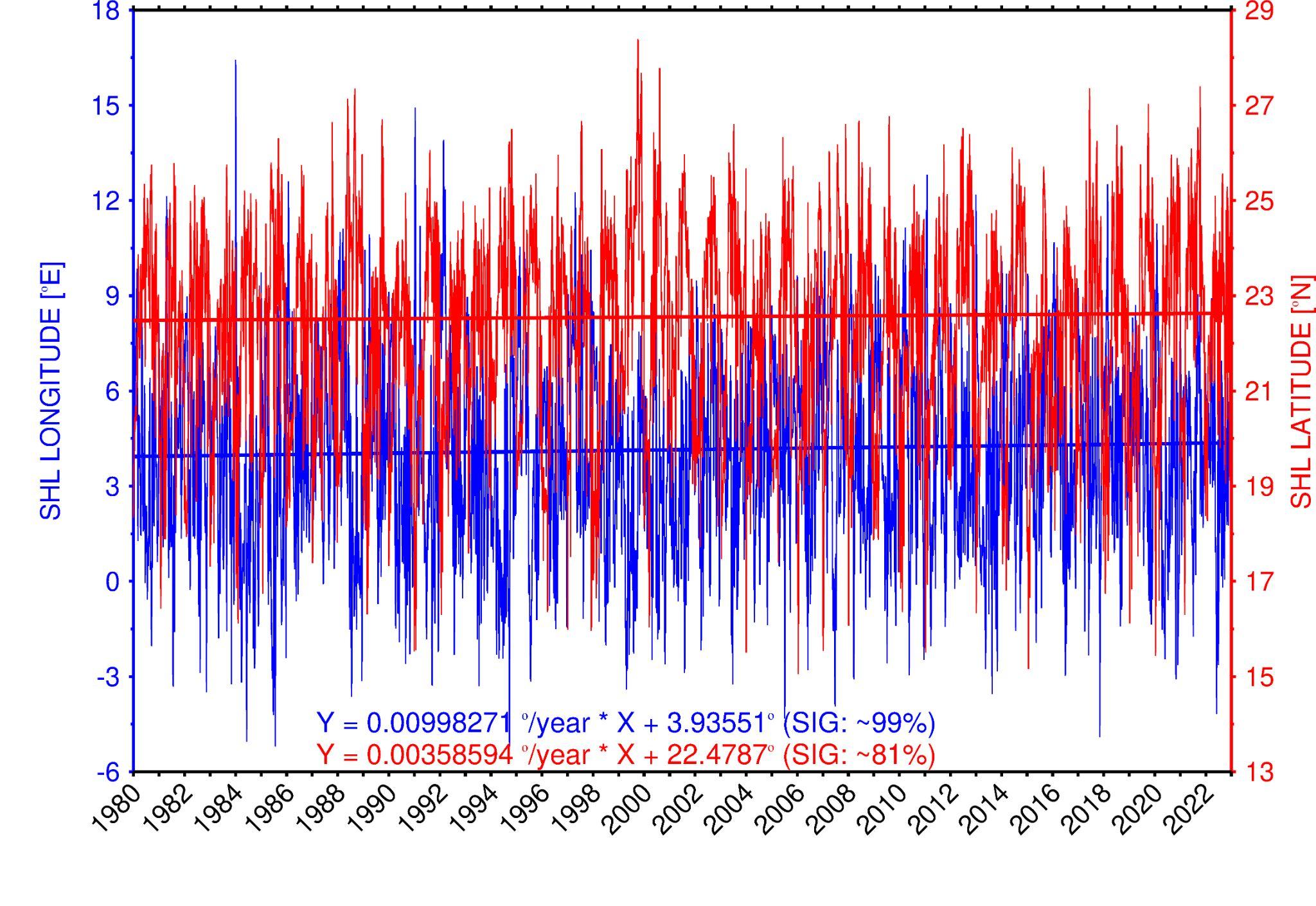 | 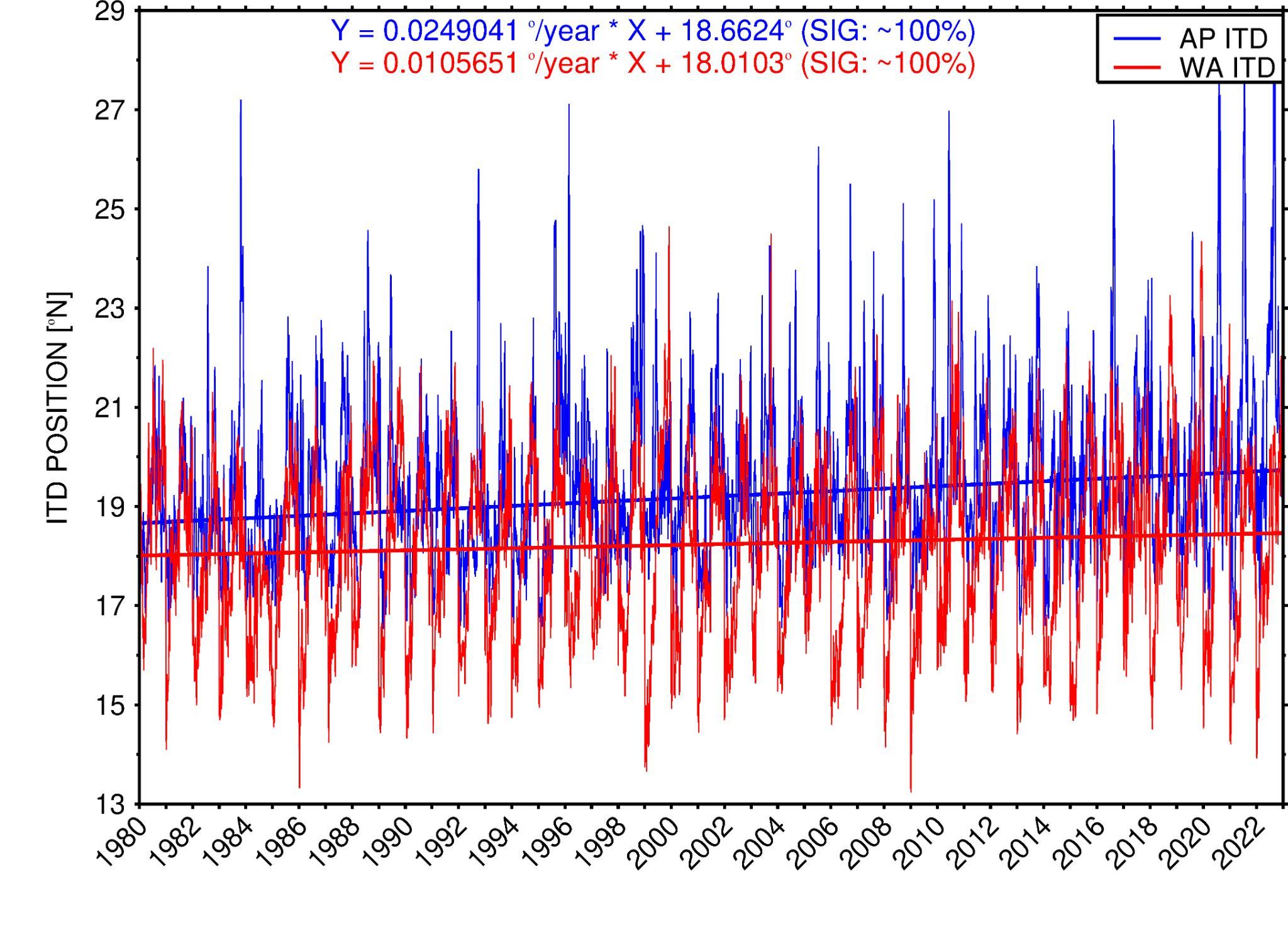 |
| **Supplementary Figure 1: Linear Regression of Heat Low and ITD:** (a) Saharan Heat Low (SHL; red) and Arabian Heat Low (AHL; blue) intensity, defined as the averaged low-level atmospheric thickness (LLAT) over the heat low regions, for JJA 1980-2022 from ERA-5. The intercept and slope are obtained with the Theil-Sen estimator while the Mann-Kendall test is used for statistical significance. (b)-(c) are as (a) but for the latitude and longitude of the AHL and SHL, respectively, defined as the LLAT-weighted latitude and longitude. (d) Averaged latitude of the Intertropical Discontinuity (ITD) over the Arabian Peninsula (43º-60ºE; red) and West Africa (10ºW-20ºE; red) for JJA 1980-2022. | |

| (a) |
| --- |
| 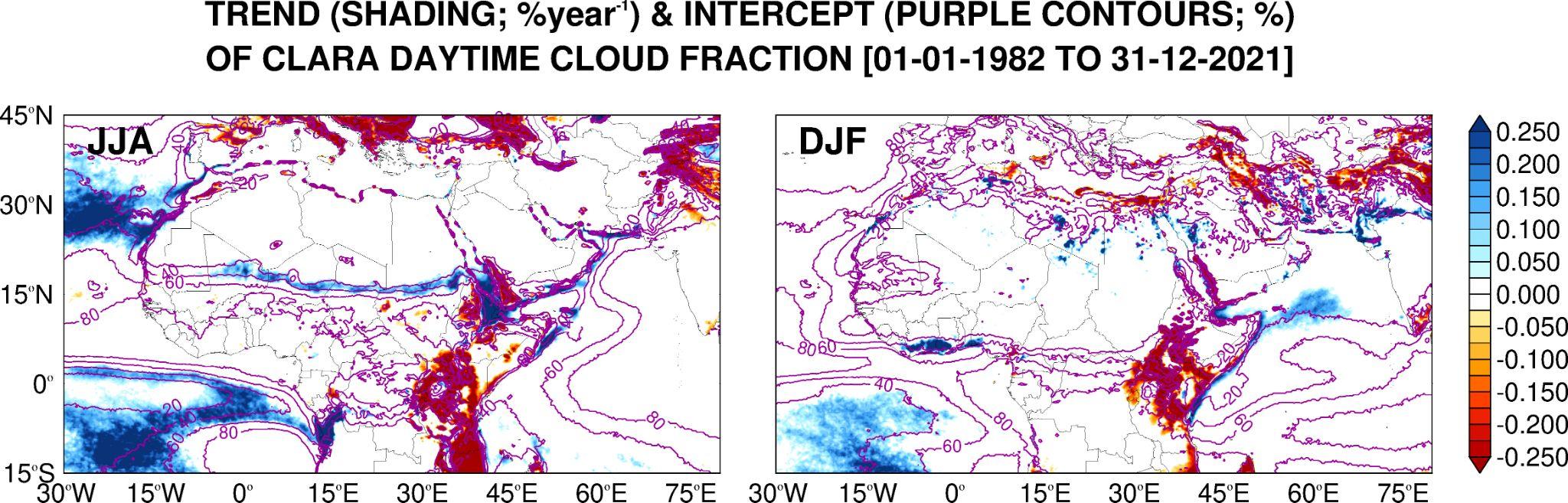 |
| (b) |
| 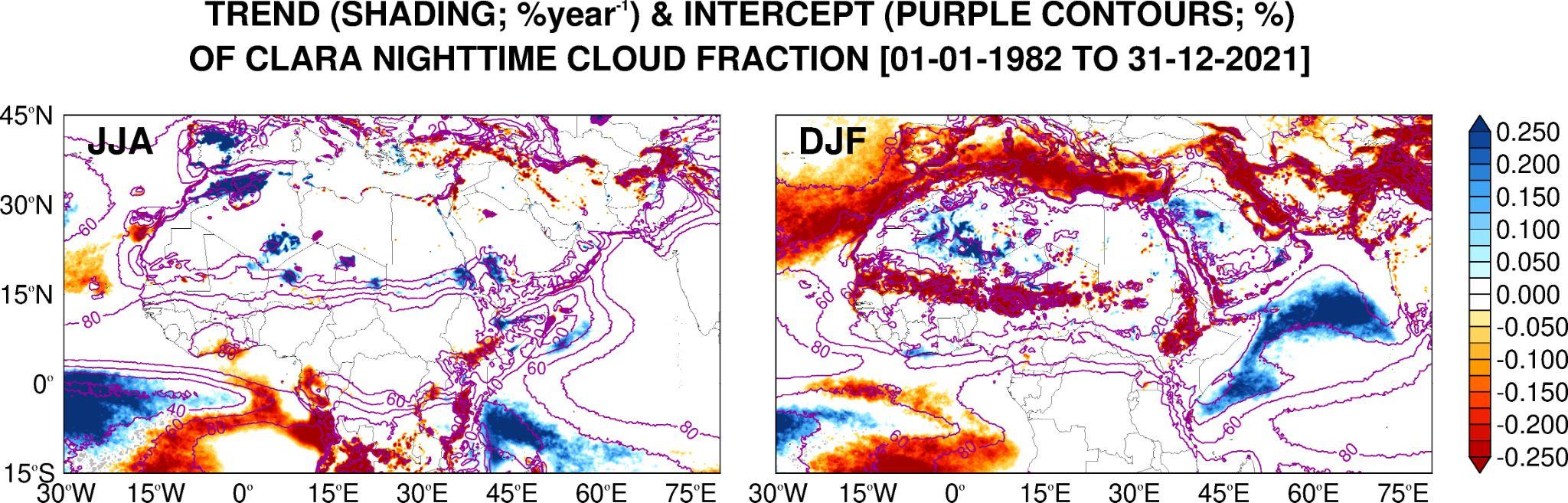 |
| **Supplementary Figure 2: Trends in CLARA Daytime and Nighttime Cloud Fraction:** Intercept (solid purple contours; %) and trend (shading; % year^-1^) of the daily-mean (a) daytime (solar zenith angle less than 75º) and (b) nighttime (solar zenith angle higher than 95º) cloud fraction from the CLARA dataset from 01 January 1982 to 31 December 2021. The intercept and slope are obtained with the Theil-Sen estimator while the Mann-Kendall test is used for statistical significance. The grey shading indicates regions where the trend is not statistically significant at the 95% confidence level. |

| (a) |
| --- |
| 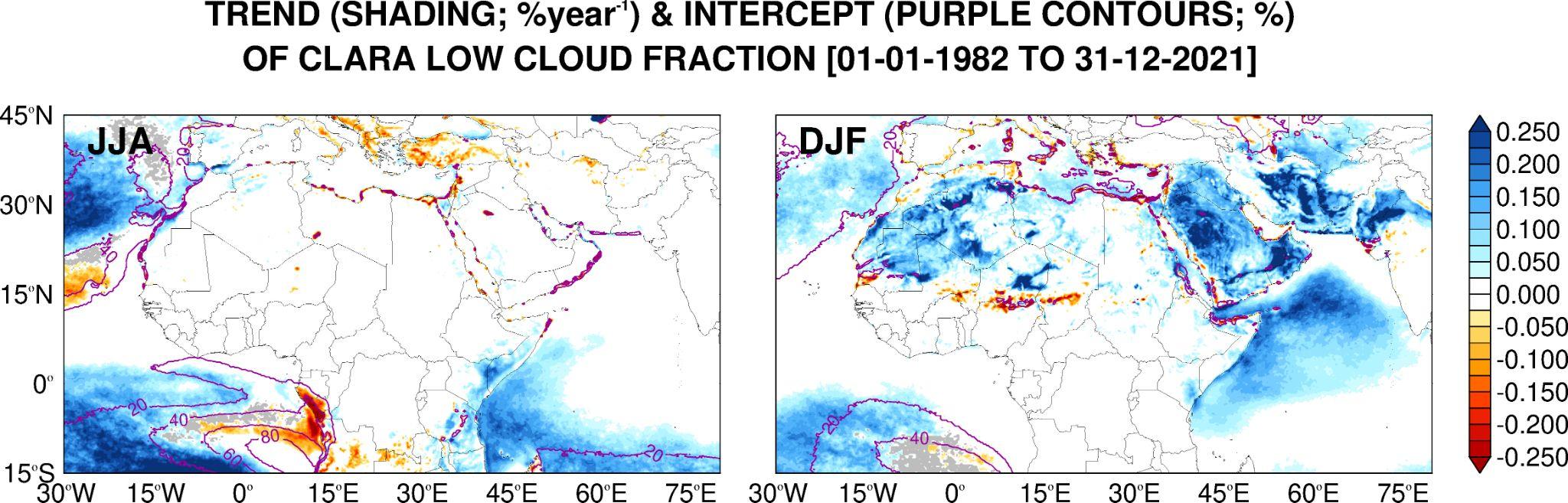 |
| (b) |
| 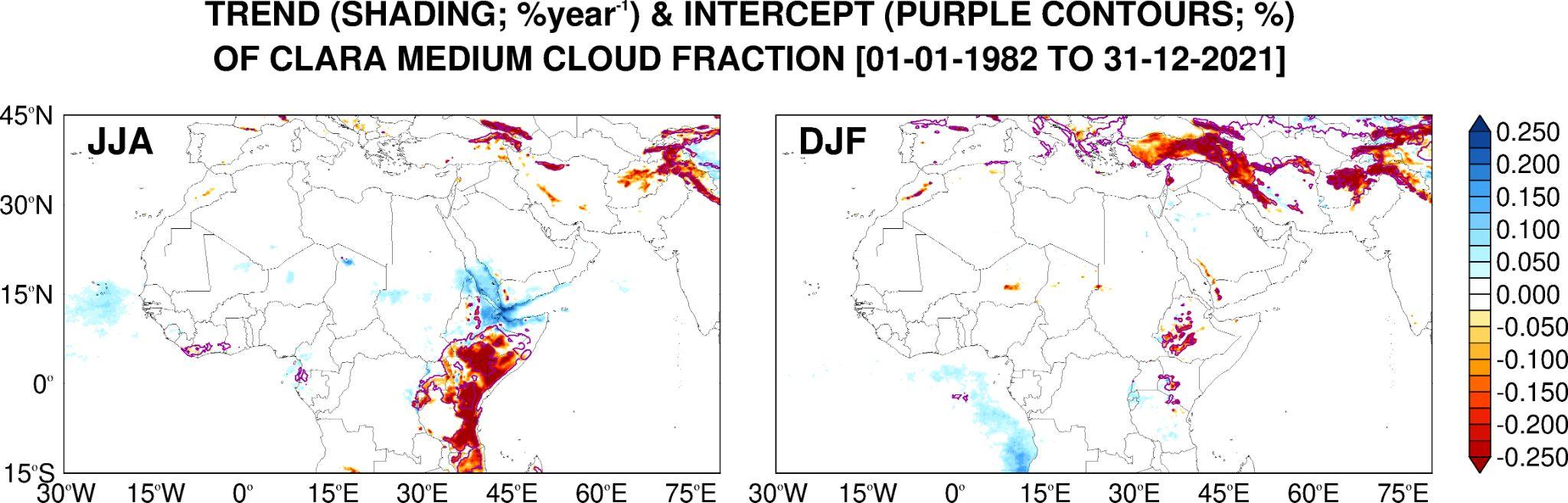 |
| (c) |
| 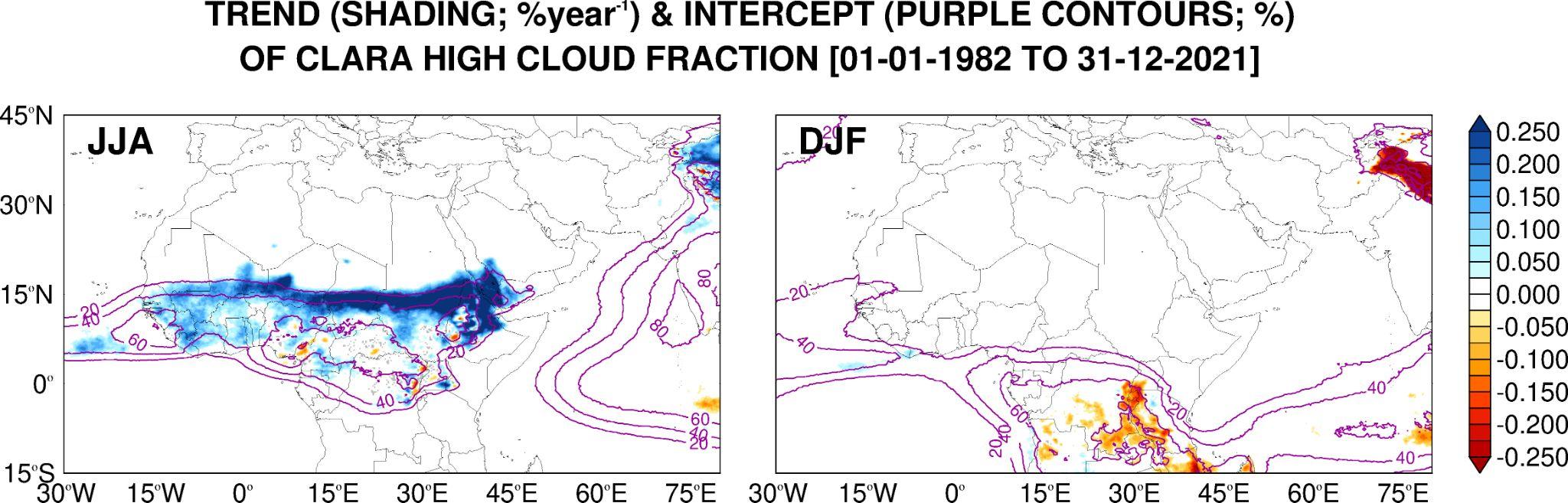 |
| (d) |
| 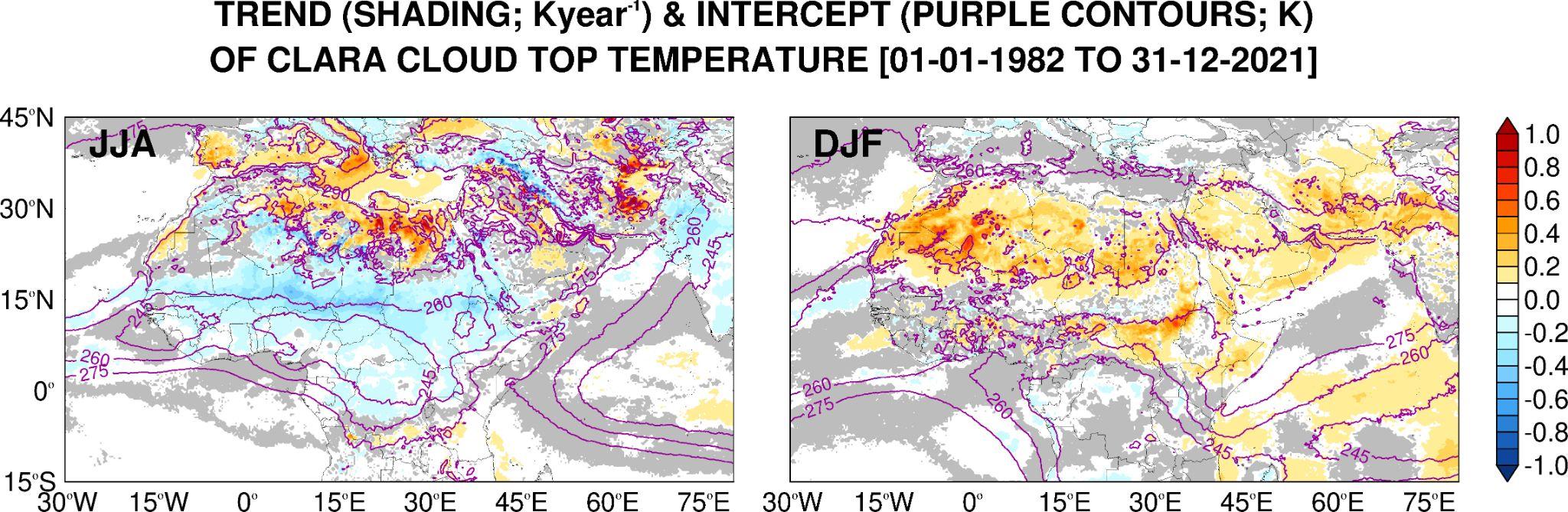 |
| **Supplementary Figure 3: Trends in CLARA Cloud Fraction and Cloud Top Temperature Fields:** Intercept (solid purple contours; %) and trend (shading; % year^-1^) of the daily-mean (a) low (clouds below 680 hPa), (b) middle (clouds between 680 hPa and 440 hPa) and (c) high (clouds above 440 hPa) cloud fraction and (d) cloud top height (K for the intercept and K year^-1^) from the CLARA dataset from 01 January 1982 to 31 December 2021. The intercept and slope are obtained with the Theil-Sen estimator while the Mann-Kendall test is used for statistical significance. The grey shading indicates regions where the trend is not statistically significant at the 95% confidence level. |

| (a) |
| --- |
| 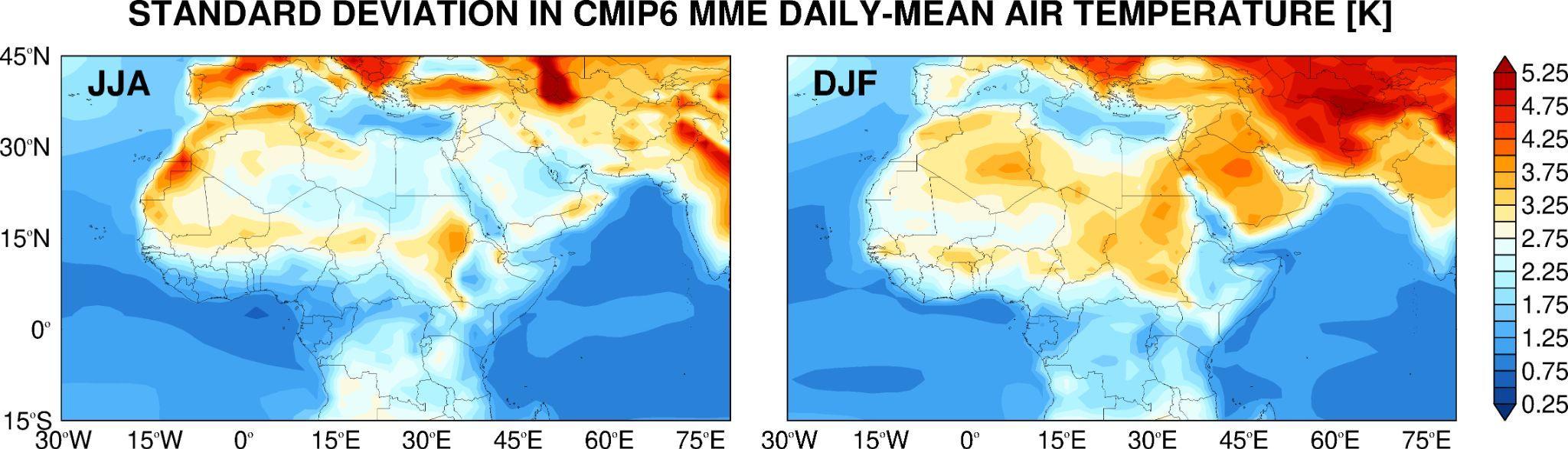 |
| (b) |
| 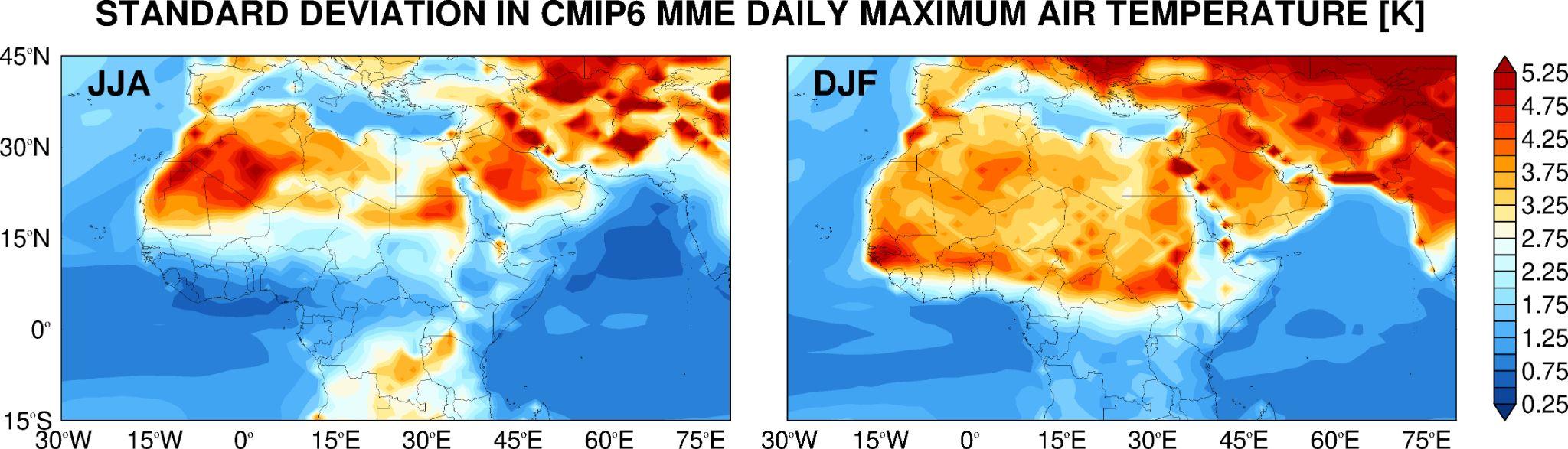 |
| (c) |
| 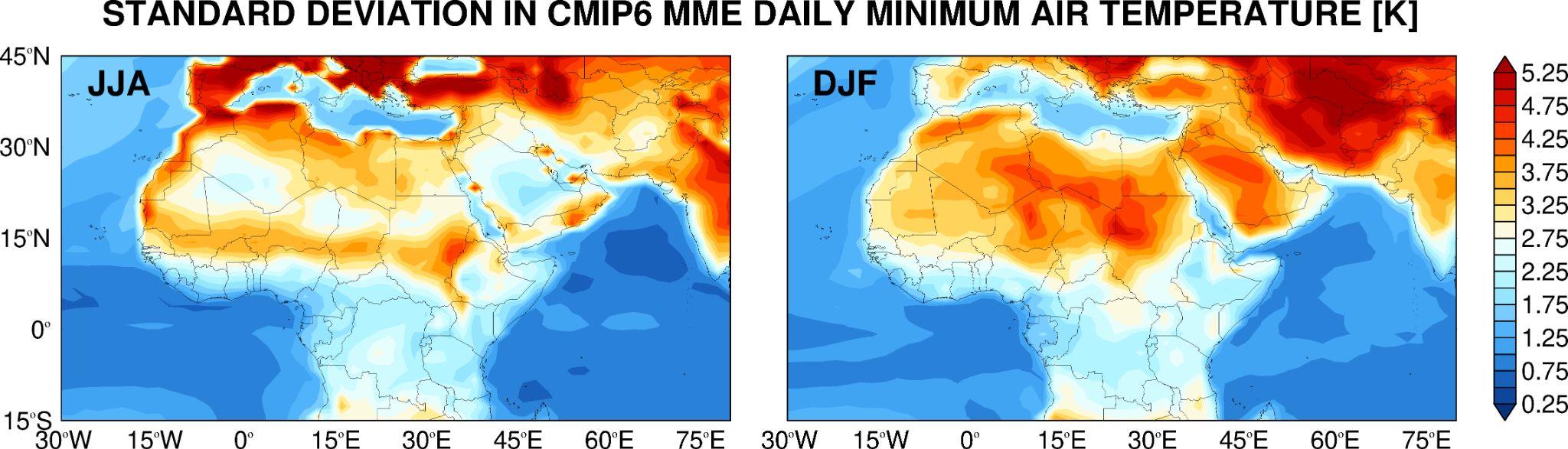 |
| **Supplementary Figure 4: CMIP6 MME inter-Model Spread for Daily Air Temperature Data**: Standard deviation of the CMIP6 MME for the (a) daily-mean and daily (b) maximum and (c) minimum air temperature (K) averaged over 1980-2014 and for the boreal summer (JJA; left) and winter (DJF; right) seasons. |
| (a) |
| 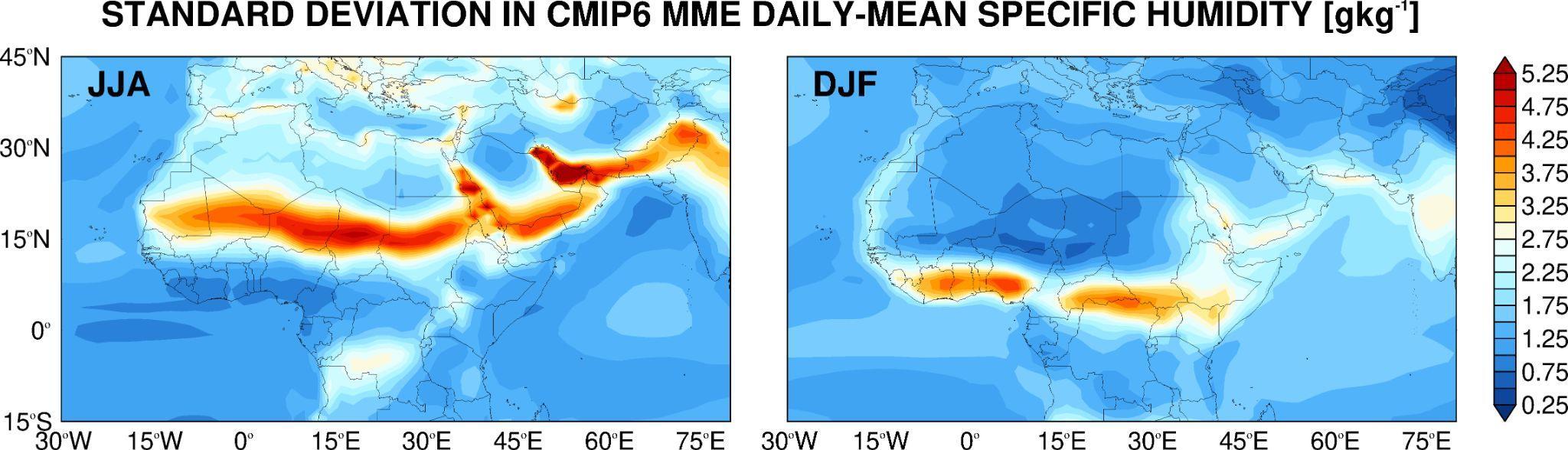 |
| (b) |
| 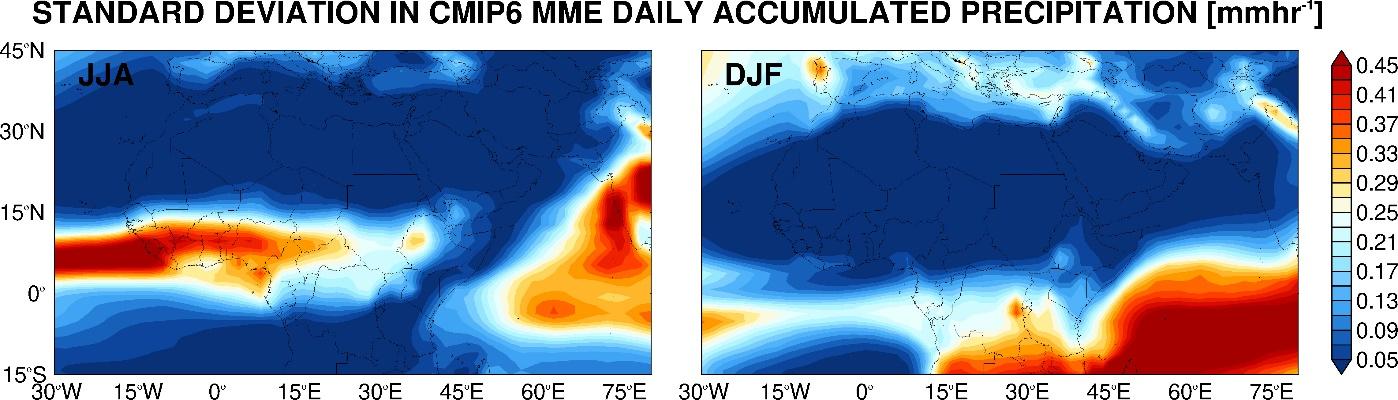 |
| (c) |
| 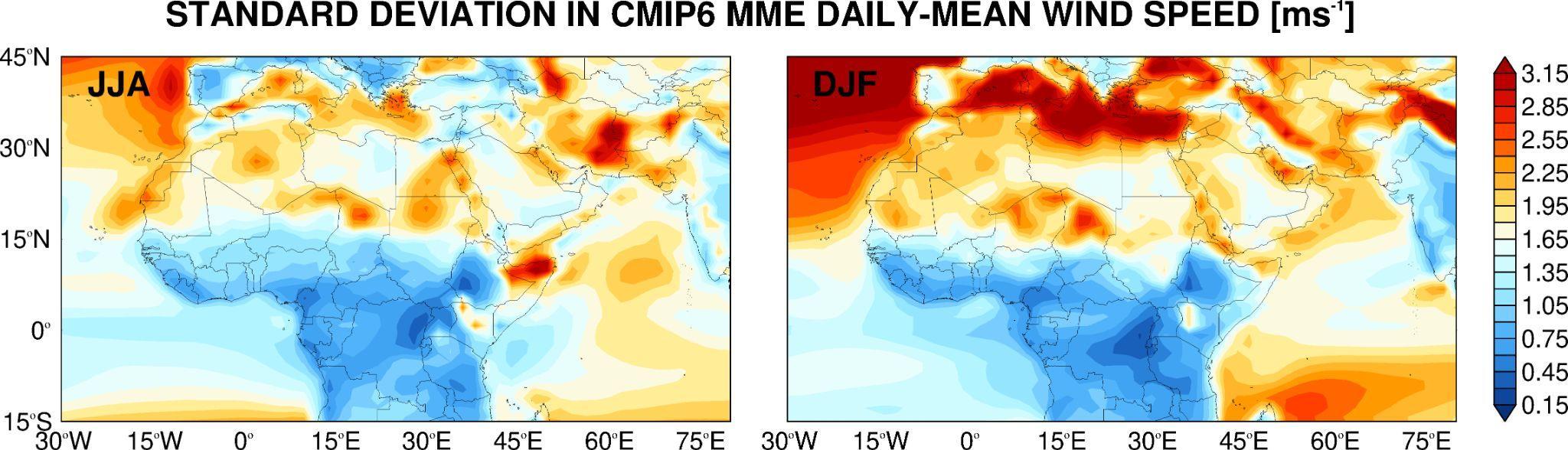 |
| (d) |
| 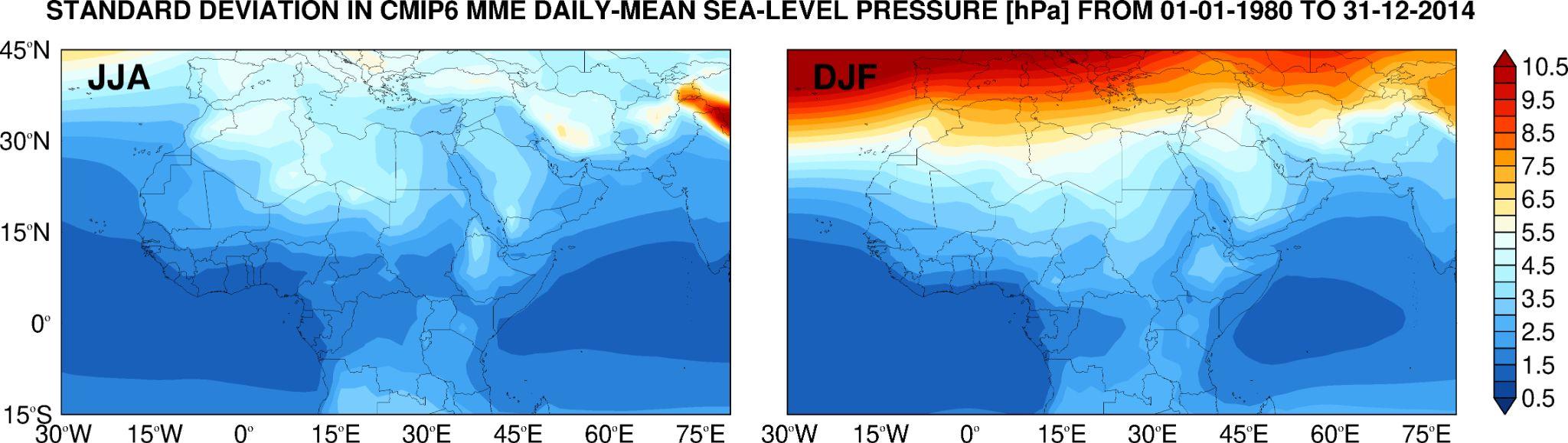 |
| **Supplementary Figure 5: CMIP6 MME Inter-Model Spread for Daily Humidity, Precipitation, Wind Speed and Sea-Level Pressure Data**: Standard deviation of the CMIP6 MME for the (a) daily-mean specific humidity (g kg^-1^), (b) daily precipitation (mm hr^-1^), and daily-mean (c) 10-m wind speed (m s^-1^) and (d) sea-level pressure (hPa) averaged over 1980-2014 and for the boreal summer (JJA; left) and winter (DJF; right) seasons. |

| (a) | (b) |
| --- | --- |
| 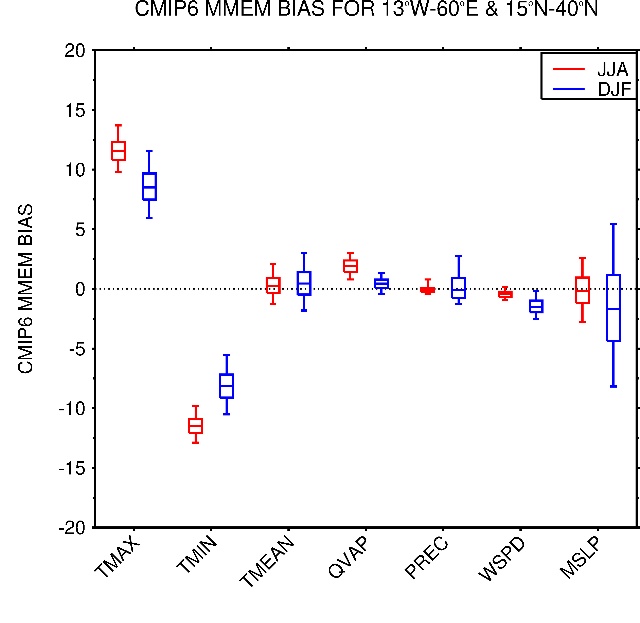 | 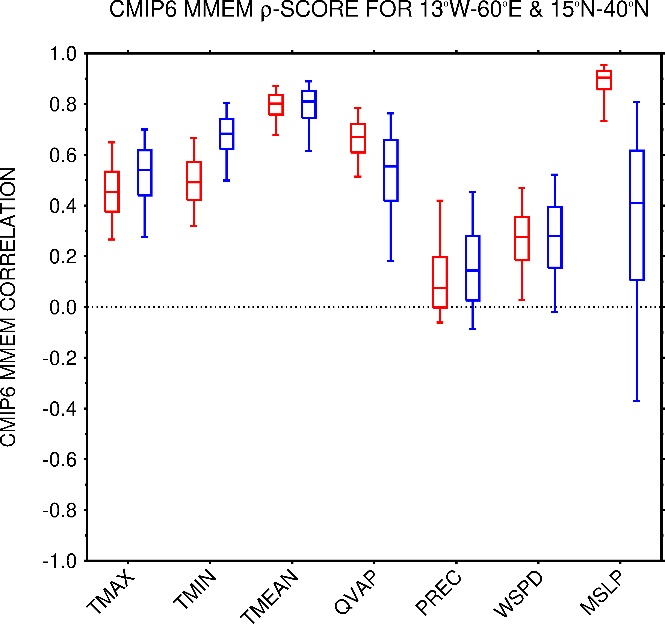 |
| (c) | (d) |
| 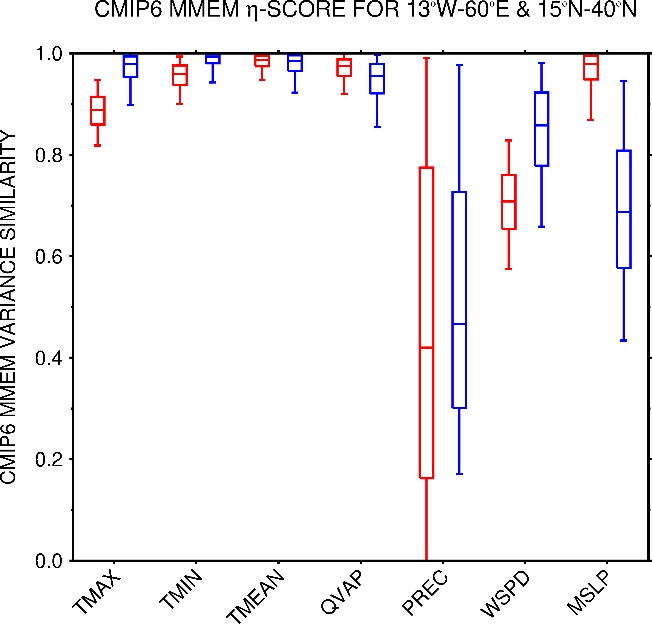 | 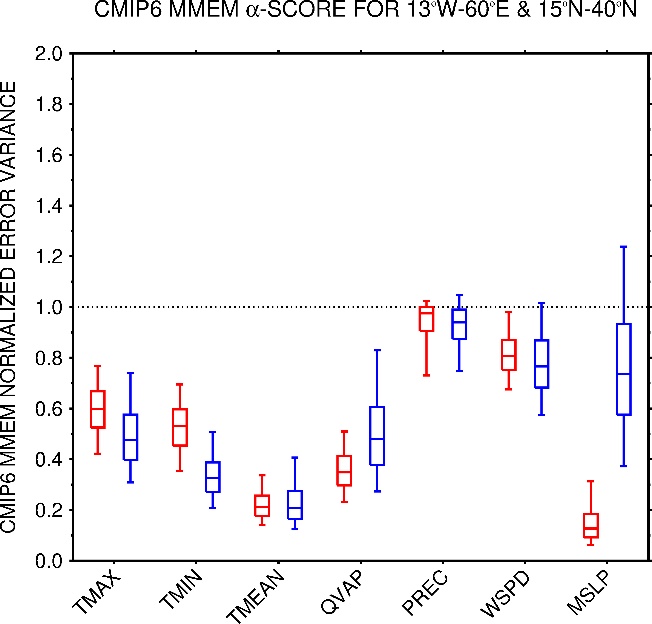 |
| **Supplementary Figure 6: Spatial Statistical Assessment of CMIP6 MME against Station Observations:** CMIP6 MME (a) bias, (b) correlation (ρ), (c) variance similarity (η) and (d) normalized error variance (α) for the daily maximum (TMAX), minimum (TMIN) and mean (TMEAN) air temperatures (bias in K), daily-mean specific humidity (QVAP; g kg^-1^), daily precipitation (PREC; mm day^-1^), and daily-mean 10-m wind speed (WSPD; m s^-1^) and sea-level pressure (MSLP; hPa) averaged over 1980-2014 at the location of 206 NOAA GSOD stations in the MENA region (13ºW-60ºE, 15º-40ºN). The scores are given for the boreal summer (JJA; red) and winter (DJF; blue) seasons. The verification diagnostics are computed as follows: for a given season and each day, the skill scores are extracted using data from all the 206 stations. The box plots display the statistics for the summer and winter seasons, giving the 5^th^, 25^th^, 50^th^ (median), 75^th^ and 95^th^ percentiles. | |

| (a) |
| --- |
| 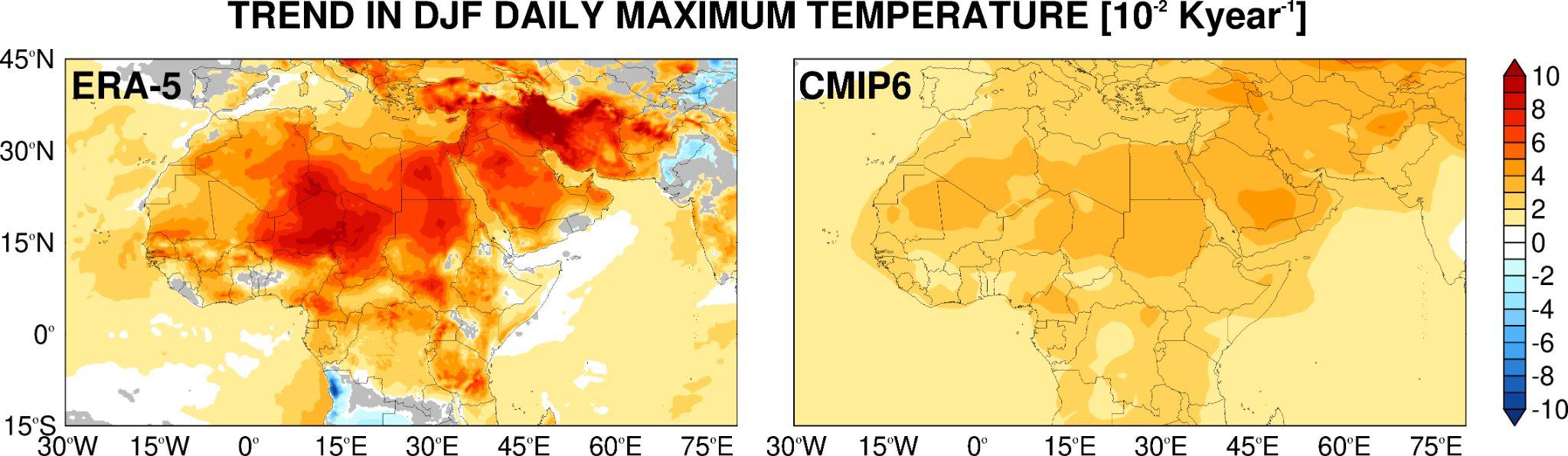 |
| (b) |
| 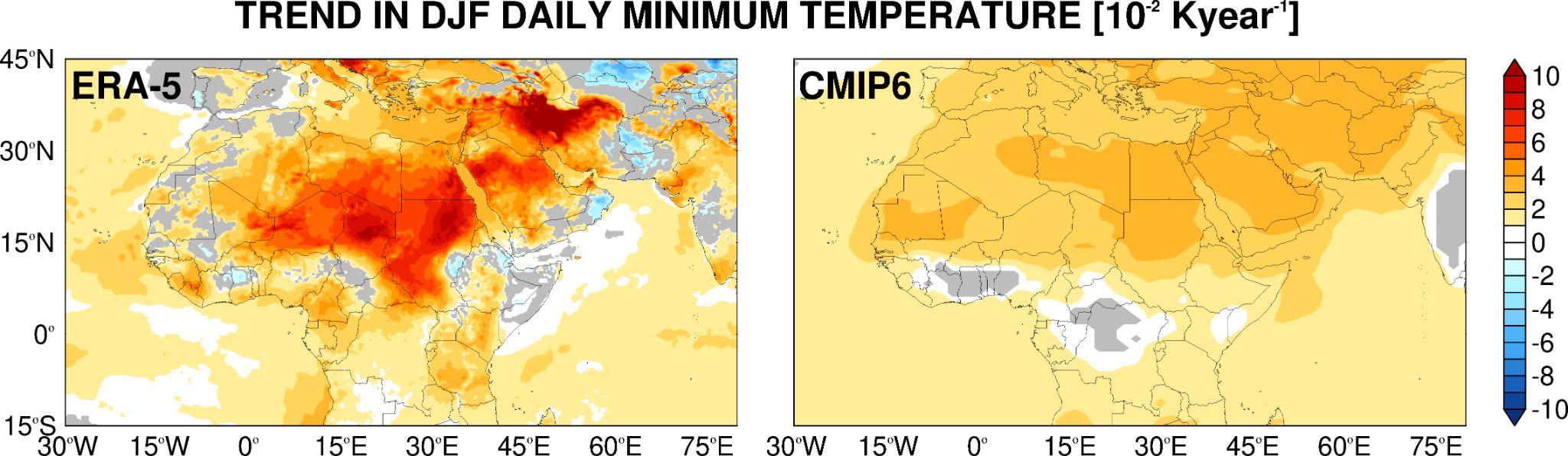 |
| (c) |
| 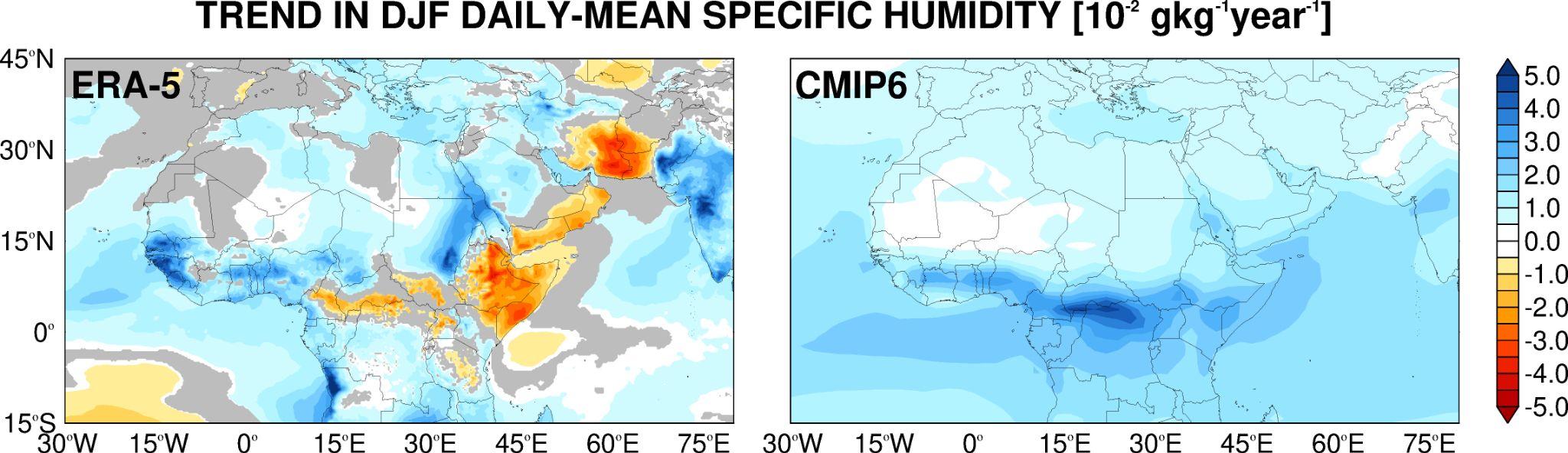 |
| **Supplementary Figure 7: Evaluation of CMIP6 MME Air Temperature and Humidity against ERA-5 Reanalysis (Boreal Winter, DJF)**: (a) Linear trend in the seasonal daily maximum temperature from ERA-5 (left) and the CMIP6 MMEM (right) for the boreal winter season for 1980-2014 (units of 10^-2^ K year^-1^). The intercept and slope are obtained with the Theil-Sen estimator while the Mann-Kendall test is used for statistical significance. Regions where the trend is not statistically significant at the 95% confidence level are shaded in grey. (b)-(c) are as (a) but for the daily minimum temperature (10^-2^ K year^-1^) and the daily-mean specific humidity (10^-2^ g kg^-1^ year^-1^), respectively. |
| (a) |
| 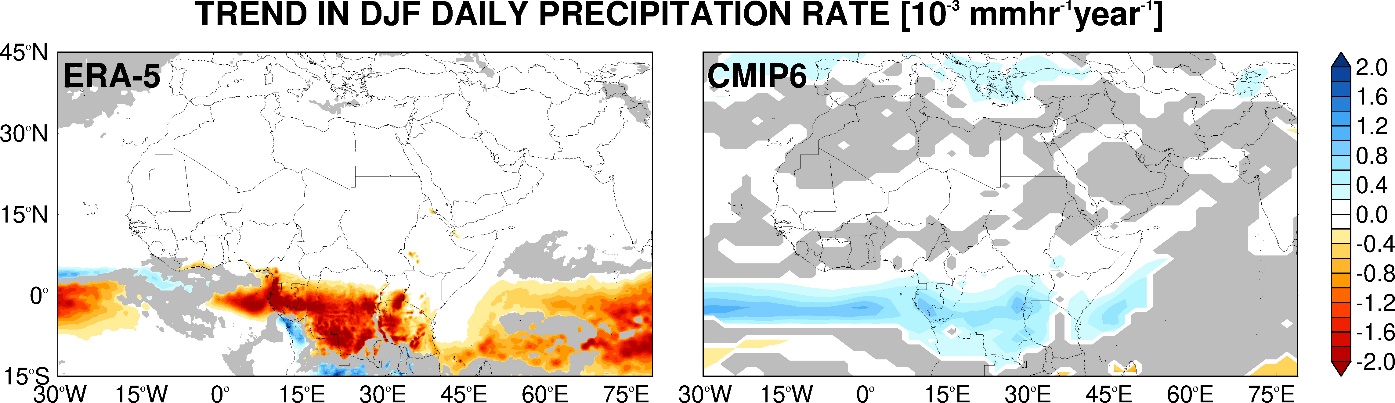 |
| (b) |
| 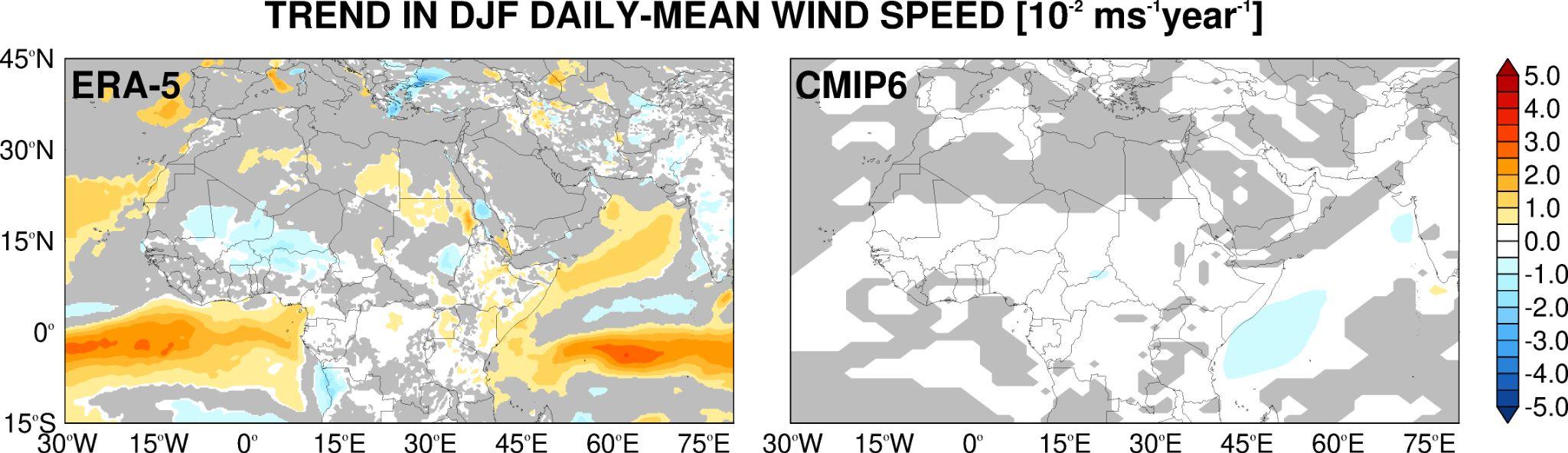 |
| (c) |
| 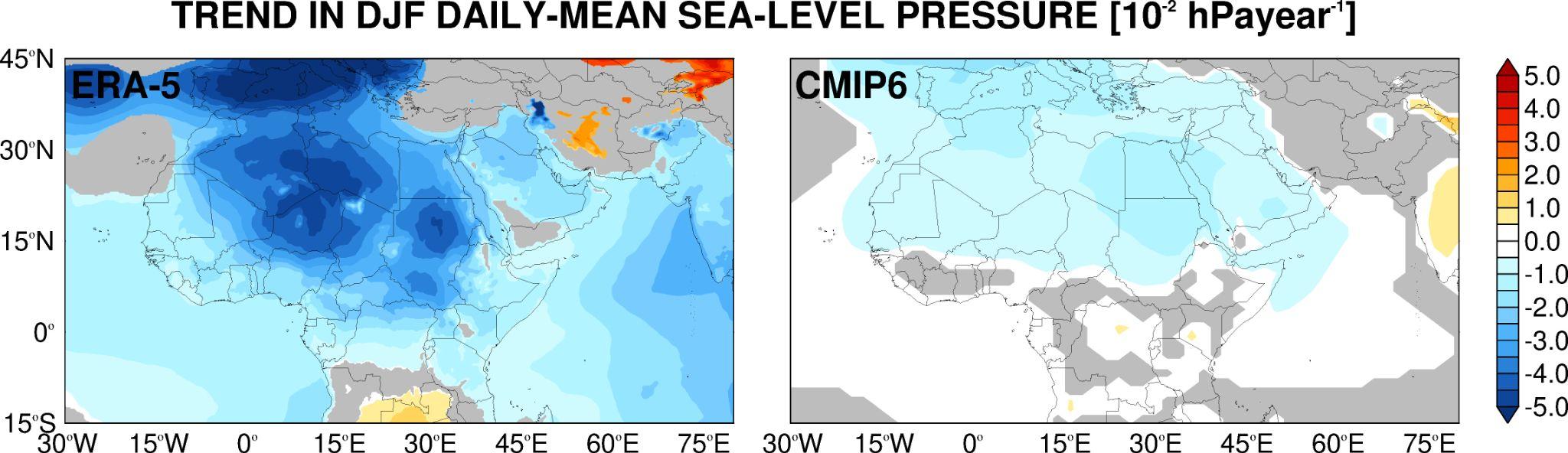 |
| **Supplementary Figure 8: Evaluation of CMIP6 MME Precipitation, Wind Speed and Sea-Level Pressure against ERA-5 Reanalysis (Boreal Winter, DJF)**: (a) Linear trend in the seasonal daily precipitation from ERA-5 (left) and the CMIP6 MMEM (right) for the boreal winter season for 1980-2014 (units of 10^-3^ mm hr^-1^ year^-1^). The intercept and slope are obtained with the Theil-Sen estimator while the Mann-Kendall test is used for statistical significance. Regions where the trend is not statistically significant at the 95% confidence level are shaded in grey. (b)-(c) are as (a) but for the daily-mean wind speed (10^-2^ m s^-1^ year^-1^) and sea-level pressure (10^-2^ hPa year^-1^), respectively. |

| (a) |
| --- |
| **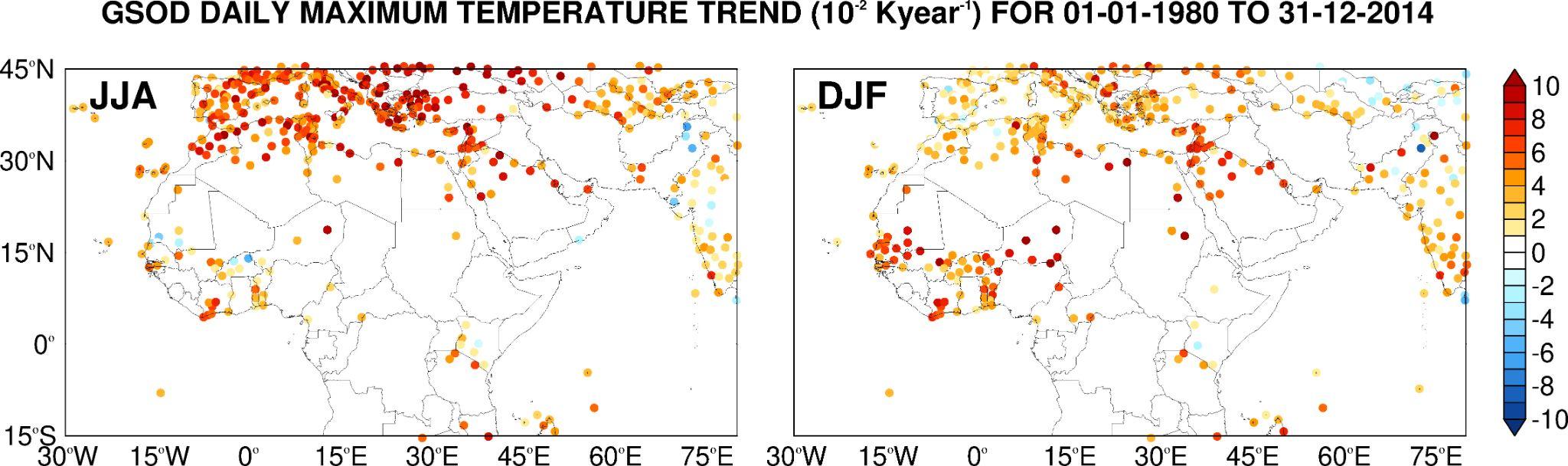** |
| (b) |
| **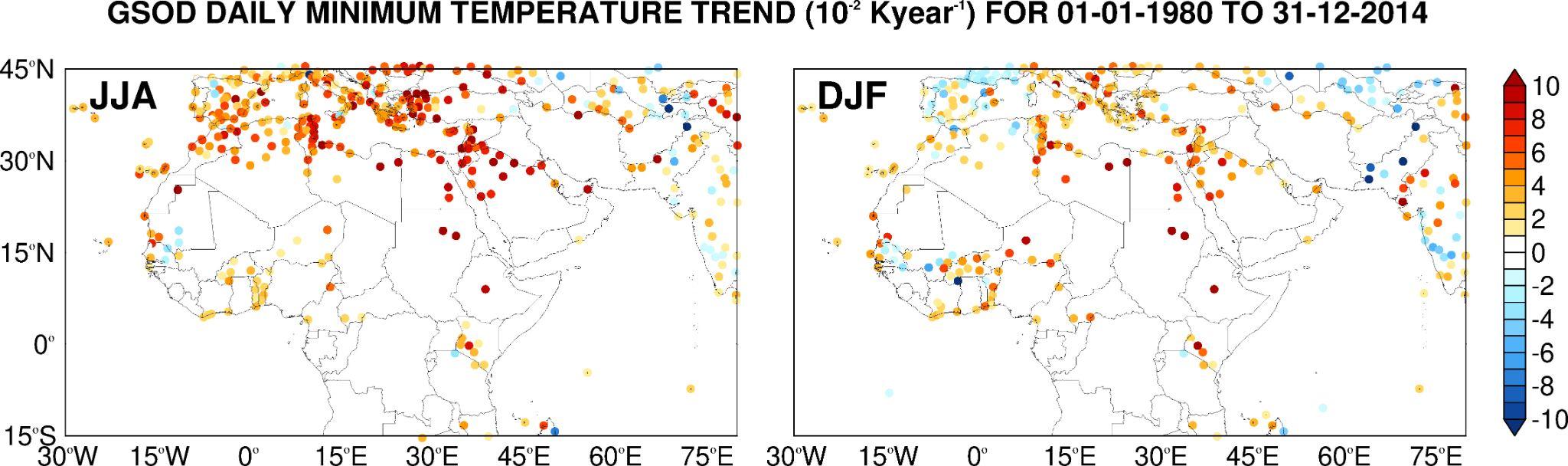** |
| **Supplementary Figure 9: Trends in NOAA GSOD Air Temperature Station Data:** Trends in NOAA GSOD (a) daily maximum and (b) minimum air temperature (10^-2^ K year^-1^) for 1980-2014. The shading gives the Theil-Sen's slopes, which are only shown if statistically significant at 95% confidence level with the Mann-Kendall test. |

| (a) |
| --- |
| **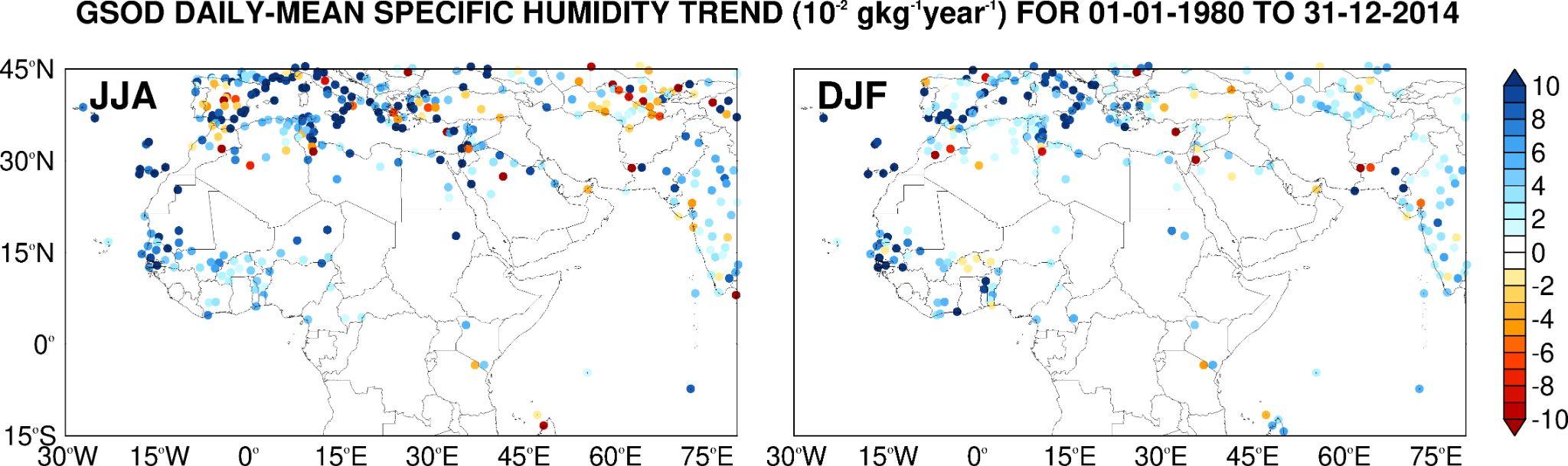** |
| (b) |
| **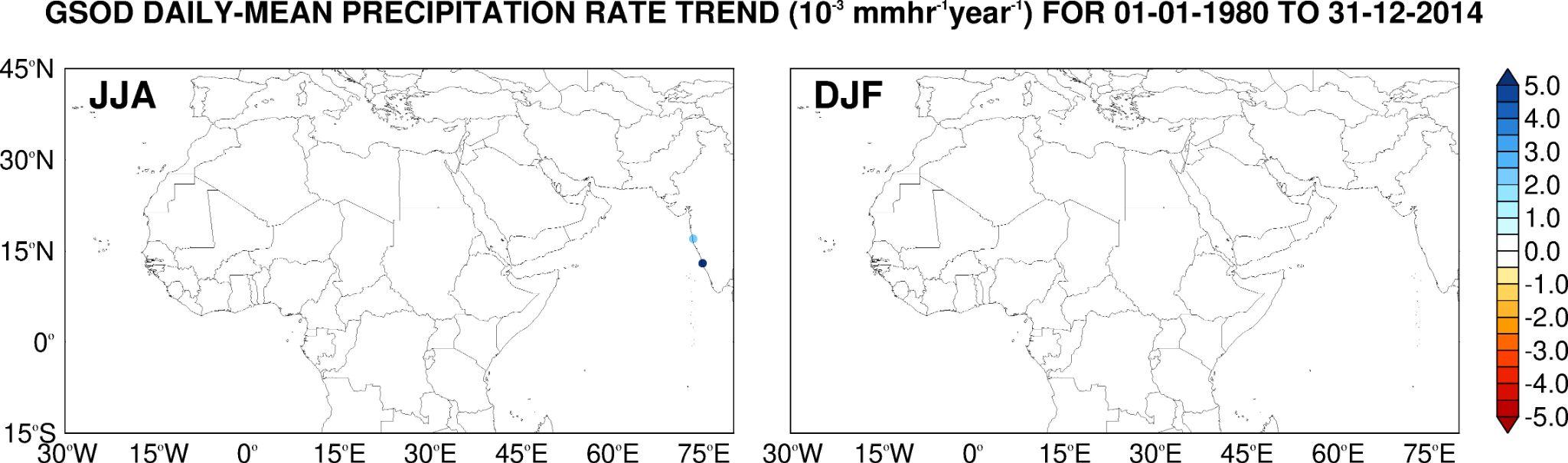** |
| (c) |
| **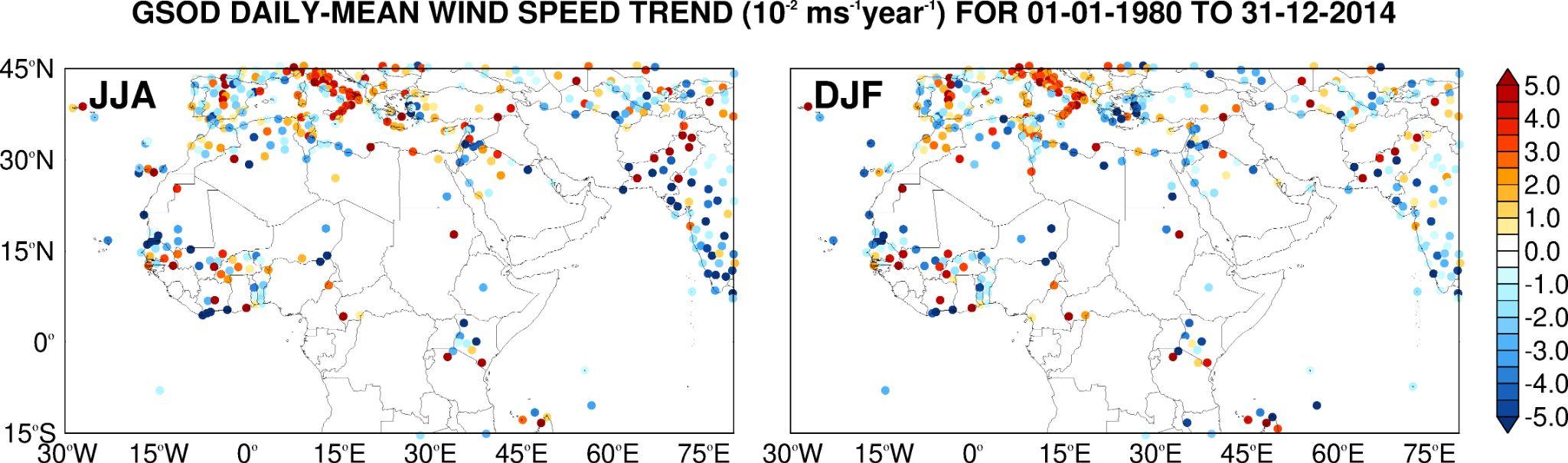** |
| (d) |
| **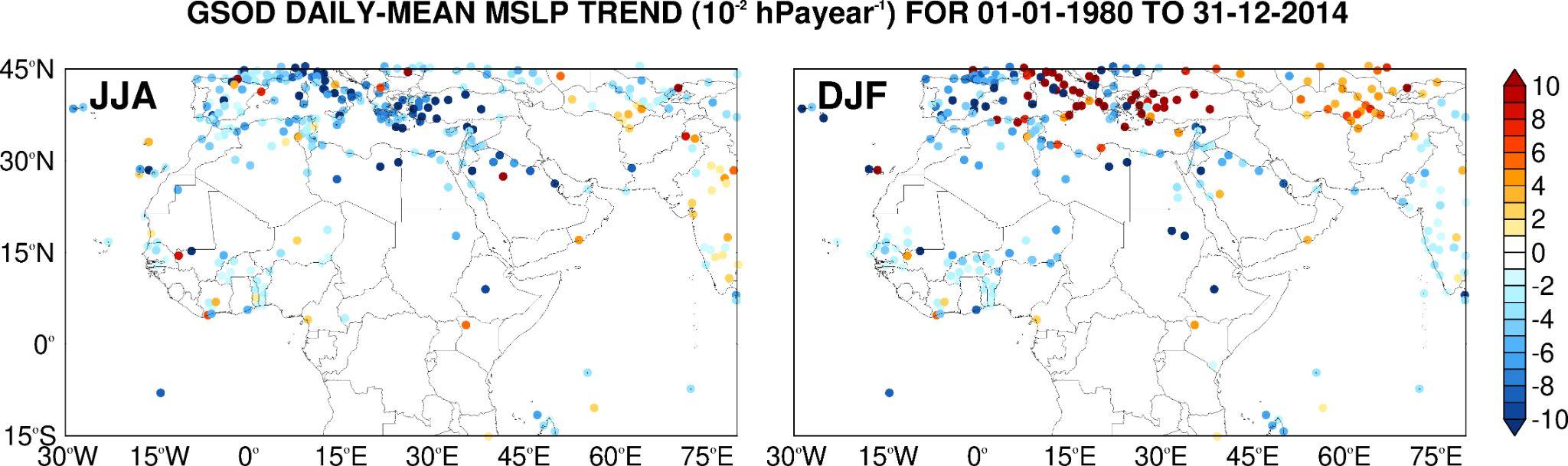** |
| **Supplementary Figure 10: Trends in NOAA GSOD Humidity, Precipitation, Wind Speed and Sea-Level Pressure Station Data:** Trends in NOAA GSOD (a) daily-mean specific humidity (10^-2^ g kg^-1^ year^-1^), (b) daily precipitation (10^-3^ mm hr^-1^ year^-1^), and daily-mean (c) wind speed (10^-2^ m s^-1^ year^-1^) and (d) sea-level pressure (10^-2^ hPa year^-1^) for 1980-2014. The shading gives the Theil-Sen's slopes, which are only shown if statistically significant at 95% confidence level with the Mann-Kendall test. |
| (a) |
| 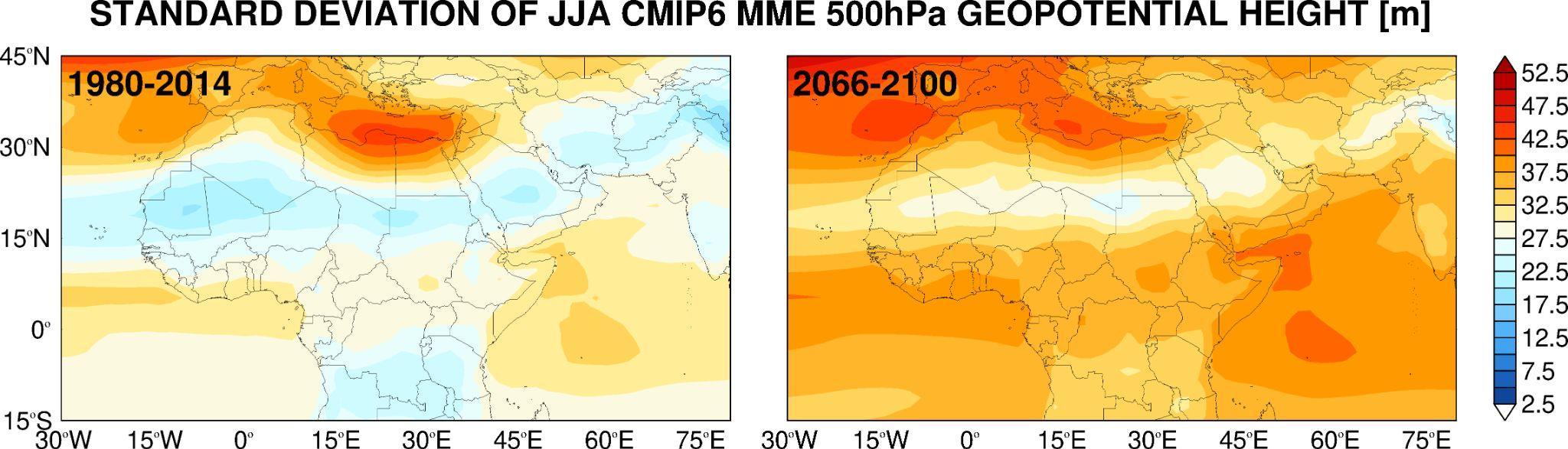 |
| (b) |
| 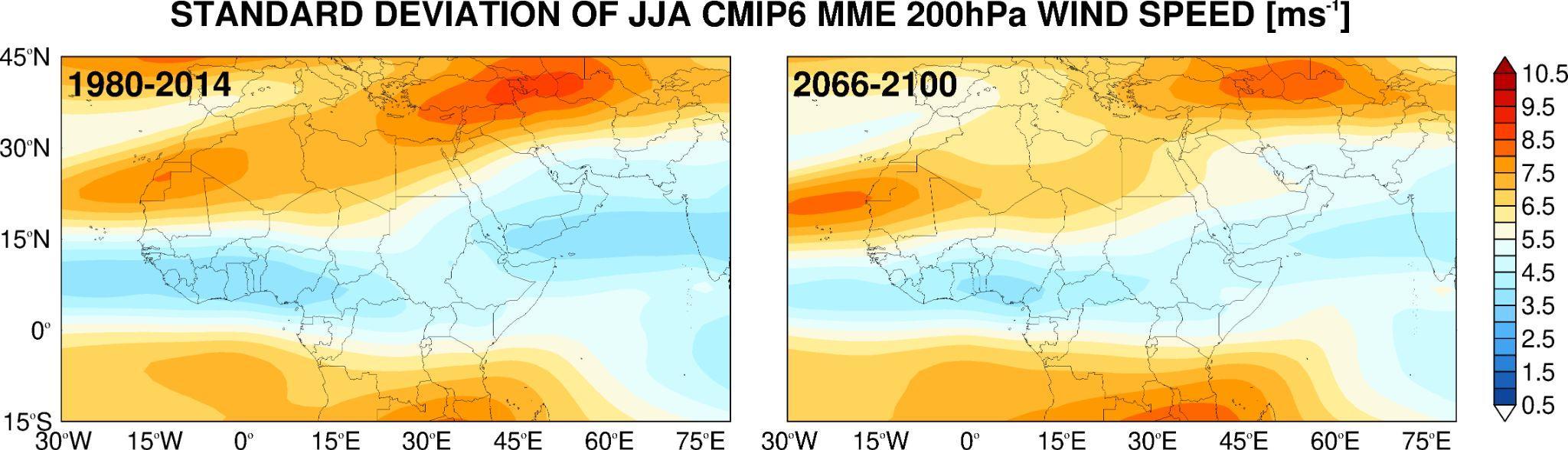 |
| (c) |
| 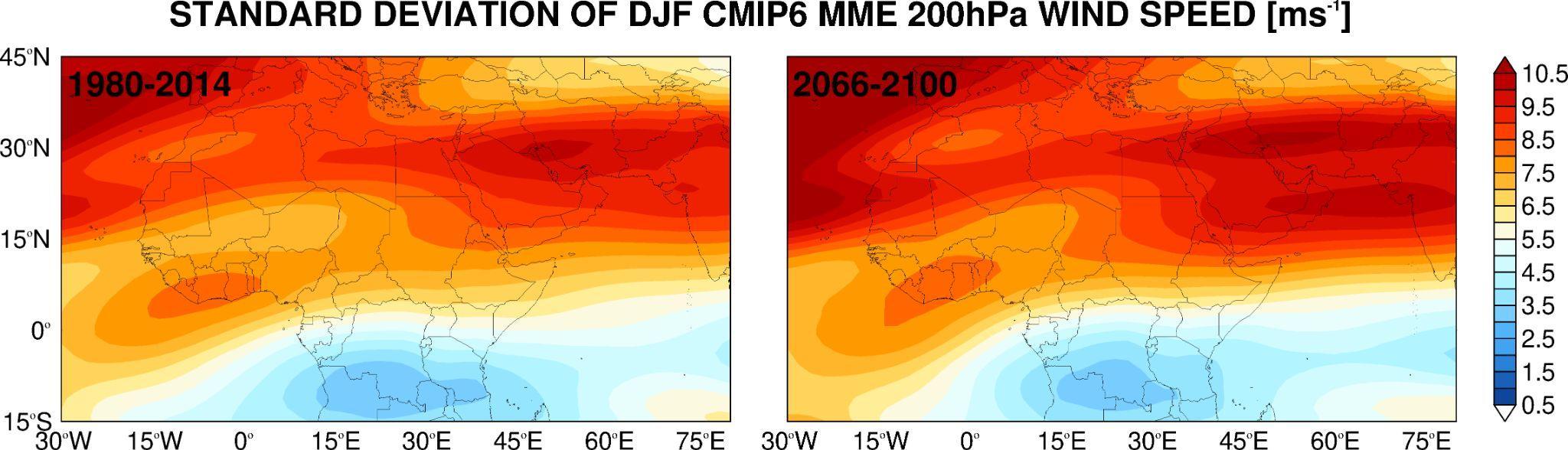 |
| (d) |
| 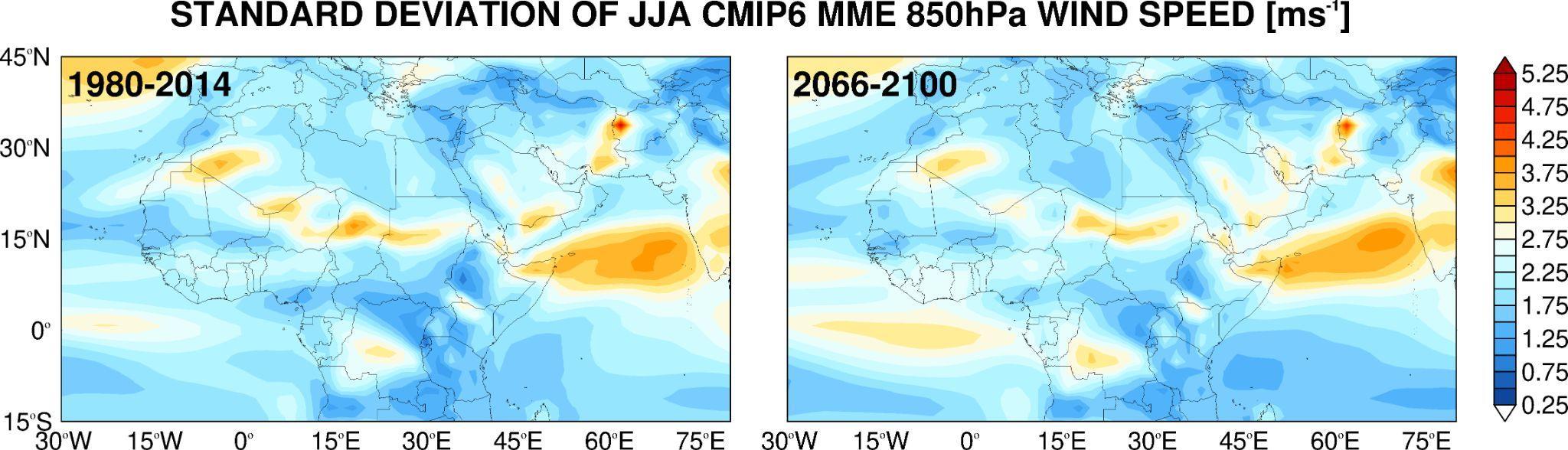 |
| (e) |
| 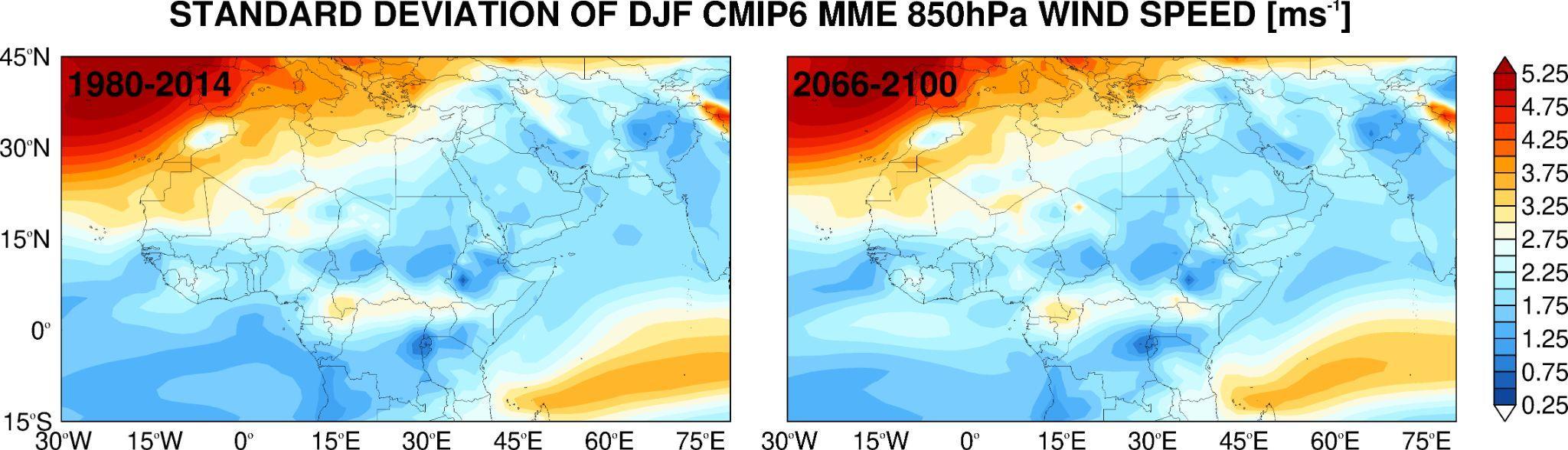 |
| **Supplementary Figure 11: CMIP6 MME Inter-Model Spread for Monthly Data**: Standard deviation of the CMIP6 MME for the monthly 500 hPa geopotential height (m) for the boreal summer for the historical period (1980-2014; left) and SSP5-8.5 climate change period (2066-2100; right). (b)-(c) and (d)-(e) are as (a) but for the summer and winter 200 hPa and 850 hPa wind speed (m s^-1^), respectively. |
